# Elucidating "lucidum": Distinguishing the diverse laccate *Ganoderma* species of the United States

**DOI:** 10.1371/journal.pone.0199738

**Published:** 2018-07-18

**Authors:** A. L. Loyd, C. W. Barnes, B. W. Held, M. J. Schink, M. E. Smith, J. A. Smith, R. A. Blanchette

**Affiliations:** 1 University of Florida, School of Forest Resources and Conservation, Gainesville, FL, United States of America; 2 The F.A. Bartlett Tree Experts Company, Charlotte, NC, United States of America; 3 Departamento Nacional de Protection Vegetal, INAP, Quito, Ecuador; 4 University of Minnesota, Department of Plant Pathology, St. Paul, MN, United States of America; 5 Independent Researcher, Port Crane, NY, United States of America; 6 University of Florida, Department of Plant Pathology, Gainesville, FL, United States of America; Ruhr-Universitat Bochum, GERMANY

## Abstract

*Ganoderma* is a large, diverse and globally-distributed genus in the Basidiomycota that includes species causing a white rot form of wood decay on a variety of tree species. For the past century, many studies of *Ganoderma* in North America and other regions of the world have used the name *G*. *lucidum* sensu lato for any laccate (shiny or varnished) *Ganoderma* species growing on hardwood trees or substrates. Molecular studies have established that *G*. *lucidum* sensu stricto (Curtis) Karst is native to Europe and some parts of China. To determine the species of the laccate *Ganoderma* that are present in the United States, we studied over 500 collections from recently collected samples and herbarium specimens from hardwoods, conifers, and monocots. A multilocus phylogeny using ITS, *tef1α*, *rpb1* and *rpb2* revealed three well-supported clades, similar to previously reported findings. From the U.S. collections, thirteen taxa representing twelve species were identified, including: *G*. *curtisii*, *G*. *lucidum* sensu stricto, *G*. *martinicense*, *G*. *oregonense*, *G*. *polychromum*, *G*. *ravenelii*, *G*. *sessile*, *G*. *tsugae*, *G*. *tuberculosum*, *G*. cf. *weberianum*, *G*. *zonatum*, and *Tomophagus colossus* (syn. *G*. *colossus*). The species *G*. *meredithiae* is synonymized with *G*. *curtisii*, and considered a physiological variant that specializes in decay of pines. The designation *G*. *curtisii* f.sp. *meredithiae* forma specialis nov. is proposed. Species such as *G*. *curtisii* and *G*. *sessile*, once considered as *G*. *lucidum* sensu lato, were found to be divergent from one another, and highly divergent from *G*. *lucidum* sensu stricto. Morphological characteristics such as context tissue color and features (e.g. melanoid bands), basidiospore shape and size, geographic location, and host preference were found to aid in species identification. Surprisingly, *G*. *lucidum* sensu stricto was found in the U.S., but only in geographically restricted areas of northern Utah and California. These collections appear to have resulted from the introduction of this species into the United States possibly from mushroom growers producing *G*. *lucidum* outdoors. Overall, this study clarifies the chaotic taxonomy of the laccate *Ganoderma* in the United States, and will help to remove ambiguities from future studies focusing on the North American species of laccate *Ganoderma*.

## Introduction

*Ganoderma* is a genus of wood decay fungi in the Polyporales and Ganodermataceae containing species that are commonly observed around the world [1, 2]. The word *Ganoderma* is Greek from *“*Gano” meaning shiny and “derma” meaning skin. *Ganoderma* was erected as a genus in 1881 by Karsten and included only one species, *G*. *lucidum* (Curtis) Karst [3]. Previously, it was characterized as *Boletus lucidus* Curtis (1781) and then *Polyporus lucidus* (Curtis) Fr. (1821) [3]. The species *P*. *lucidus* was characterized by having a laccate pileus and stipe, which Murrill suspects was the reason for Karsten’s division because only one species was included, *G*. *lucidum* [4]. Patouillard revised Karsten’s genus *Ganoderma* to include all species with pigmented spores, adhering tubes and laccate crusted pilei, which resulted in a total of 48 species classified under the genus *Ganoderma* in his 1889 monograph [4, 5]. Until Murrill investigated *Ganoderma* in North America in 1902, previous work had focused solely on European species including *G*. *lucidum*, *G*. *resinaceum* Boud. (1890) and *G*. *valesiacum* Boud. (1895) [4, 6, 7].

Murrill described 17 new *Ganoderma* species in his treatises of North American polypores [4, 7], including *G*. *oregonense* Murrill from *Picea sitchensis* in Oregon, *G*. *sessile* Murrill occurring on decaying wood of deciduous trees widespread in the eastern United States (U.S.), *G*. *tsugae* Murrill from decaying trunks, stumps and roots of *Tsuga canadensis* in the boreal hemlock forests of the eastern U.S., *G*. *tuberculosum* Murrill occurring on dead wood in Central America and *G*. *zonatum* Murrill from decayed palm wood in Florida [4, 7]. Subsequent reports on the genus in North America were contradictory, and there was little agreement regarding the species designated by Murrill [1, 8–12]. For example, many studies focusing on North American taxa considered *G*. *sessile* as a synonym of the European species *G*. *lucidum* [8, 11]. This was based on similar fruiting body morphology and host preference for both *G*. *sessile* and *G*. *lucidum*, in which they were both laccate with reddish-brown crust, with or without a stipe, and fruiting on decaying hardwood substrates [7, 8, 11]. Similarly, *G*. *tsugae* was also synonymized with *G*. *lucidum*, despite the former being described from conifers and the latter from deciduous trees [8]. Atkinson [8] erected *G*. *subperforatum* G.F. Atk. in 1908 to include taxa that had “smooth” basidiospores, which were due to fine echinulations protruding through the perisporium (hyaline outer wall) of the double-walled basidiospore. Although Atkinson also considered *G*. *tsugae* a synonym for *G*. *lucidum* [8], Overholts accepted the species *G*. *tsugae* due to its temperate distribution, darker pileus and pure white context tissue [11]. Both Atkinson & Overholts accepted *G*. *curtisii* (Berk.) Murrill as a unique species that occurs in more southern latitudes of the U.S. [8, 11]. Although no data were reported, in 1920 in an update on Polyporaceae of North America, Murrill conceded that *G*. *sessile* was closely related to the European *G*. *lucidum* [13]. It is difficult to determine if this concession was the result of other mycologists’ scrutiny or from a scientific foundation, due to the retention of the name by Murrill in a later publication [14] and a single herbarium collection in 1926 (FLAS-F-08907). In addition, Murrill also described *G*. *sessiliforme* Murrill from dead wood in Mexico [15]. The brief description of *G*. *sessiliforme* described the fruiting body as similar to *G*. *sessile*, but with smaller dimensions. A decade later, Haddow [9] considered *G*. *sessile* as a unique taxon, and suggested Atkinson’s *G*. *subperforatum* was a synonym of *G*. *sessile*, on the basis of the “smooth” spores compared to the “rough” spores of *G*. *lucidum*, which was the original basis for *G*. *subperforatum* when erected earlier by Atkinson. Furthermore, Haddow [9] agreed with Atkinson that *G*. *tsugae* is a synonym of *G*. *lucidum* and should not be its own species, despite the previous work of Overholts [11]. Until this point, all identifications of *Ganoderma* taxa were based on fruiting body morphology, geography, host, and spore characters [4, 7–9, 12].

Nobles [10, 16] characterized the cultural characteristics of numerous wood-inhabiting Hymenomycetes including *Ganoderma* species. Her work essentially laid the foundation for culture-based identifications of the Basidiomycota [16]. Although Nobles recognized *G*. *lucidum* in her 1948 publication [10] as the correct name for the taxon from North American isolates that produce numerous broadly ovoid to elongate smooth chlamydospores (12.0–21.0 x 7.5–10.5 μm), she corrected this misnomer in 1965 by amending the name to *G*. *sessile* [16]. Nobles recognized that there were differences in cultural characteristics between *G*. *oregonense*, *G*. *sessile*, and *G*. *tsugae* [10, 16]. Clarifying further, Bazallo & Wright as well as Steyaert agreed with Haddow’s distinction between *G*. *lucidum* and *G*. *sessile* on the basis of having “smooth” spores, but they synonymized *G*. *sessile* with *G*. *resinaceum*, a previously described European taxon [12, 17].

In the monograph of “North American Polypores” [1] written in 1986, which is still the only comprehensive treatise on this group of fungi in North America, Gilbertson and Ryvarden recognize five laccate species; *G*. *colossum* (Fr.) CF. Baker, *G*. *lucidum*, *G*. *oregonense*, *G*. *tsugae*, and *G*. *zonatum*. In the comments for *G*. *lucidum* the authors discuss the taxonomic perplexity of this particular species complex, and left it somewhat unresolved. The culture morphology (e.g. presence of chlamydospores) of the North American *G*. *lucidum* sensu lato recognized by Adaskaveg & Gilbertson [6] was indistinguishable and biologically compatible with the European species *G*. *resinaceum*, where monokaryons of North American *G*. *lucidum* sensu lato could be mated with monokaryons of *G*. *resinaceum*. A similar conclusion was drawn by Bazallo & Wright as well as Steyaert, where they suggest that *G*. *sessile* and *G*. *resinaceum* are taxonomic synonyms [12, 17]. Biological species concepts are not always appropriate for designating species ranks for fungi [18]. Although intersterility groups can be successful in laboratory tests, these successful pairings may be due to geographic distributions of a shared common ancestor, and the extant taxa are in the process of allopatric speciation, or vicariance, due to a lack of gene flow because of geographical barriers (e.g. mountains, oceans, etc.) [18, 19].

Adaskaveg & Gilbertson [20] erected *G*. *meredithiae* Adask. & Gilb. as a distinct species occurring on pines in the Gulf Coast of the United States. This species was distinguished from the five species found in Gilbertson & Ryvarden’s polypore treatise [1], by its host restriction to pines, slow cultural growth rate, no chlamydospores, plumose or feathery culture morphology, and frequently lobed or branched pilocystidia [20]. Melanoid deposits in the context tissue, which Steyaert suggested was a distinguishing feature for some *Ganoderma* taxa, are present in *G*. *meredithiae* [12, 20]. Although this species was circumscribed, in the same paper the authors informally suggest that *G*. *curtisii* and the North American *G*. *lucidum* sensu lato were synonyms [20].

With the rise of molecular phylogenetic analyses in the late 20^th^ century, species concept hypotheses were tested to determine the relatedness amongst the nuanced morphological variabilities of the laccate *Ganoderma* taxa. Moncalvo et al. [21] constructed a phylogeny of the rDNA, and found six major clades amongst the 29 samples in the analysis. Samples labeled as *G*. *lucidum* were found in five of the six clades [21]. Hong and Jung [22] found similar results, and showed that *G*. *resinaceum* from Europe, and the North American *G*. *lucidum* (which Adaskaveg and Gilbertson found to be biologically compatible *in vitro*) were sister taxa and were also more closely related to each other than *G*. *lucidum* sensu stricto [6, 21]. In addition, the authors showed that the North American taxa *G*. *tsugae* and *G*. *oregonense* were closely related and that these two species were relatives of the European taxon *G*. *valesiacum* Boud. From this they determined that *G*. *carnosum* Pat., *G*. *oregonense*, *G*. *tsugae*, and *G*. *valesiacum* were conspecific, and ranked them to the *G*. *valesiacum* species complex, where all taxa are restricted to temperate coniferous forests [21, 22]. However, it is hypothesized that the laccate *Ganoderma* species share a common tropical ancestor since more species diversity has been documented from tropical locations [21, 23].

Furthermore, early rDNA phylogenies of global collections of *Ganoderma* taxa showed that morphological features and cultural characteristics appeared to be highly polyphyletic [21, 22, 24]. While morphology is often polyphyletic in many groups of fungi, it is difficult to determine the validity of the polyphyletic nature of the morphological characters in the laccate *Ganoderma* taxa due to the use of ambiguous species names. Recent surveys of *Ganoderma* taxa conducted in the neotropics have revealed a diversity of *Ganoderma* species including several novel, phylogenetically supported species [25–28]. Most notably, Welti & Courtecuisse [28] described *G*. *martinicense*, which is a close relative of the Asian taxon *G*. *multipileum* Ding Hou. *Ganoderma multipileum* has recently been identified from tropical Asian locations as one of the cultivated *Ganoderma* species used in traditional medicine that was once labeled as *G*. *lucidum* sensu lato [29, 30]. Furthermore, the species *G*. *sessiliforme* has never been reported in the U.S., but Torres-Torres et al. [27] recently collected it in geographically restricted areas of Mexico, near the type location, along with *G*. *sessile*, which was more rare.

Finally, a recent multilocus phylogeny, using ITS, *tef*, *rpb1*, and *rpb2*, revealed that the global diversity of the laccate *Ganoderma* species included three supported major lineages [2]. These results agree with several of the earlier works focusing mostly on morphology, geography and host preference showing genetic affinity of *G*. *resinaceum* and *G*. *sessile*, but with statistical support separating the European and North American taxa [2]. Similarly, *Ganoderma curtisii* and *G*. *sessile* were separated with high levels of statistical support, and not considered synonyms. In addition, the phylogeny supported the similarities between *G*. *tsugae* and *G*. *oregonense* revealing that these two taxa are sister to one another, and the closest relatives of the European taxon *G*. *lucidum* sensu stricto [2]. The phylogenetic species concept using a multilocus approach is currently the most robust and accepted method for designating species ranks for the fungi [18].

The confusion surrounding the taxonomy of the North American laccate *Ganoderma* species has made drawing clear inferences on ecology and biology difficult or impossible. Molecular work is needed to clarify and redefine the laccate *Ganoderma* species both globally and regionally. Specific molecular phylogenies of local taxa would allow testing of hypotheses of various phylogeographic and ecological questions. To our knowledge a comprehensive survey of the species diversity of the genus *Ganoderma* in the United States using molecular phylogenetic techniques to validate species names has not been done. The objectives of this research were to I) to identify the laccate *Ganoderma* species present in the United States using over 500 collections, II) determine relationships between the phylogenetically supported species with a multilocus analysis, and III) identify diagnostic characters for the laccate *Ganoderma* species in the U.S.

## Materials and methods

### Fungal material examined

Basidiomata representing laccate (varnished or shiny) *Ganoderma* species were collected from dead and declining trees from throughout the United States by the authors or citizen scientists. In addition, selected herbarium collections from the University of Florida (Fungal Herbarium of the University of Florida: FLAS) and North Carolina State University (North Carolina State Collection Larry Grand: NCSCLG) herbaria were morphologically examined and used for this study.

Isolations from basidiomata were attempted for all fresh collections by taking small pieces (<1 mm^3^) of context tissue with a sterile scalpel and placing onto 2% malt extract agar (MEA) (Difco Laboratories, Franklin Lakes, NJ) according to the manufacturer’s instructions with the addition of streptomycin (100 mg/l), benomyl 95% (4 mg/l), and lactic acid (2 mL/l) to limit bacterial growth. Pure cultures were made by subculturing original isolations on MEA (without antibiotics), and grown at 28° C in the dark. Cultures were maintained on MEA slants (without antibiotics) as working stocks, and infested agar discs were submerged in sterile water for long term storage as done previously [31]. Culture collections are archived at the Center for Forest Mycology Research (CFMR) Culture Collection and Herbarium, USDA Forest Service, maintained by the Northern Research Station and housed in the Forest Products Laboratory, USDA-Forest Service in Madison, Wisconsin. Representative basidiomata collected from this study have been accessioned into the FLAS collection.

### Basidiomata morphology and host substrate classification

Morphological assessments were similar to that from Zhou et al. [2]. Color standards from Ridgway [32] were used to describe macromorphological features of the basidiomata. The color and texture of the context tissue was observed visually and described. Other contextual features such as melanoid deposits or concentric zones were noted. Basidiospores were visualized on slides in 5% KOH under 1000x magnification using a Nikon Eclipse 55i light microscope (Melville, NY), and photographed with a Canon Rebel T3i (Huntington, NY). Measurements were made using ImageJ software (www.imagej.net) for at least ten basidiospores of three to five representative collections for each species, except *Tomophagus colossus* (Fr.) Murrill, which had measurements from two basidiomata from the same collection (255FL). The size of the basidiospore width extends the entire width at the widest point from the outer wall and the length extends from the base of the basidiospore to the length of the truncated apex. Basidiospore measurements (i.e. length, width, spore shape index, and Q-ratio) were analyzed statistically in JMP PRO 12 (SAS, Cary, NC) using analysis of variance and Tukey’s HSD means separations. In addition to basidiospores, slide mounts of the context tissue and mycelium from cultures were made to note presence or absence of chlamydospores. If chlamydospores were present, at least ten were measured under 1000x magnification as described previously. In addition to morphology, information on location and host was recorded if available. Host substrate species, when available, were identified visually by the authors, or by collectors of the various specimens. From this data, hosts were broken down into major groups including cactus, conifer, cycad, hardwood, or monocot.

### DNA extraction, PCR, and sequencing

DNA was extracted from the context tissue of the basidiomata or mycelium from cultures of each accession with the Extract-N-Amp rapid DNA kit (Sigma-Aldrich, St. Louis, MO) a Qiagen DNeasy Plant Mini Kit (Qiagen, Hilden, Germany) according to the manufacturer’s instructions, or a CTAB protocol as described previously [33]. Initially, samples were identified based on morphology, host, and geographic locations, and these identifications were confirmed through sequencing the internal transcribed spacer (ITS) region of the ribosomal DNA (rDNA). ITS sequences were queried across sequences deposited in GenBank. In addition, *tef1α*, *rpb1*, and *rpb2* were sequenced for representative isolates for each species. These regions were selected to make comparisons to a recently published global phylogeny of the members of the *G*. *lucidum* species complex [2].

The ITS region was amplified with primers ITS1F and ITS4b or ITS4 as done previously [34, 35]. For the *tef1α*, *rpb1*, and *rpb2* loci primer pairs EF1-983/EF1-2218R [36], RPB1-2.2f/RPB1-Cr [37, 38], and fRPB2-5F/bRPB2-7R2 [39, 40] were used respectively and followed PCR protocols following Blanchette et al. [33]. For problematic samples, primers were designed from alignments made from *Ganoderma* sequences downloaded from GenBank for each of the loci (*tef1α*, *rpb1*, and *rpb2*). Multiple primer pairs were made for each locus, and tested for efficiency using a gradient PCR. The following primers were chosen and used for PCR amplification: *tef1α*: EF-Gano23F (5’ GGTGTCAGGCAGCTCATYGT) and EF-Gano887R (5’ CGAACTTGCARGCGATGTG), *rpb1*: RPB1-Gano18F (5’ GCGTGGTGAAATGGGGGGCT) and RPB1-Gano958R (5’ GCAACTGCTCGAACTCGTTG), *rpb2*: RPB2-Gano53F (5’ AAYTGGGGAGACCAGAA) and RPB2-Gano829R (5’ CGCCTTYAAYCGAGC). For each PCR reaction, the following reagents were used: 12.5 μl of Immomix Red Master Mix (Bioline, London, UK), 8.5μl of PCR-grade H_2_O, 1μl BSA (3% w/v), 1μl of each primer, and 1ng/μl of DNA template. Reactions were performed on a MJ Mini thermocycler (BioRad, Hercules, CA) with the following thermocycler conditions: cycle of 95° C for 10 min and followed by 35 cycles of 94° C for 30 sec, variable annealing temperatures of 55° C (ITS) 62 C (*tef1α* and *rpb1*) 53.2° C (*rpb2*) for 30 sec, and 72° C for 1 min, followed by a final extension step of 72° C for 5 min, and then 4° C. Amplicons were purified with Exo-SAP-IT (ThermoFisher, Waltham, MA) according to the manufacturer’s recommendations. Sanger sequencing was performed using both forward and reverse primers at the Interdisciplinary Center for Biotechnology Research (ICBR) at the University of Florida, Genewiz (www.genewiz.com), or the University of Minnesota Genomics Center. Forward and reverse sequences for each sample were aligned and visually edited using Geneious 10 (www.geneious.com, [41]). All sequences generated were deposited and accessioned in the GenBank sequence database.

### Phylogenetic analyses

ITS, *tef*, *rpb1*, and *rpb2* sequences for representative isolates generated from this study and reliable voucher sequences from Zhou et al. [2] were aligned for each locus using the MAFFT [41] plugin in Geneious 10, and visually edited to remove any ambiguities and minimize differences of each alignment that could have resulted from sequencing error. The alignments consisted of 98 individual sequences with 521 nucleotides for ITS, 58 individual sequences with 764 nucleotides for *tef1α*, 61 individual sequences with 615 nucleotides for *rpb1*, and 36 individual sequences with 570 nucleotides for *rpb2*. Visually edited alignments of each locus were used for independent phylogenetic analyses using the RAxML [42] plugin in Geneious 10 using a general time reversal (GTR) evolutionary model with rapid boostrapping and 1000 bootstrap replications. Trees were assessed visually to determine any incongruences between topologies across individual locus-based phylogenies. All tree topologies of the four loci were congruent, so a concatenated alignment with all four loci representing 2470 characters was used in phylogenetic analyses using RAxML and Mr. Bayes [43] plugins in Geneious 10. The RAxML analysis of the concatenated alignment used a GTR evolutionary model using rapid bootstrapping with 1000 bootsrap replications, and the Mr. Bayes analysis used a GTR evolutionary model with a gamma rate variation using four gamma categories, 1,100,000 chain length with 4 heated chains using a burn-in length of 100,000. In both analyses, *Tomophagus colossus* isolates UMNFL110 and TC-02 were used to root the tree as the outgroup, because it is a closely-related genus in the Ganodermataceae [2]. Alignments have been deposited on TreeBase and found under the submission ID 22399 (http://purl.org/phylo/treebase/phylows/study/TB2:S22399?x-access-code=a01554a96d36b989fd14482c059b6b82&format=html).

### *Ganoderma* species DNA barcoding

Although ITS has been widely recognized as the fungal barcode of life [44], it is now accepted that ITS alone can underestimate the total number of species in biodiversity studies [45]. Alternative or additional DNA barcode loci should be sought after for *Ganoderma* species, so that rapid and accurate species diagnosis can be achieved. To determine the most appropriate barcode locus for the laccate *Ganoderma* species in the U.S., RAxML trees from the aforementioned analyses for each of the four loci were visually compared to the putative species tree constructed from the concatenated alignment. Scores were given for each analysis of individual loci and the concatenated (four loci) trees. They were scored on total number of well supported terminal clades and the associated summed bootstrap values for each well-supported terminal clade. These analyses were conducted as described previously using the RAxML plugin with rapid bootstrapping and 1000 bootstrap replication in Geneious 10. Alignments were deposited on TreeBase found under the submission ID 22399 (http://purl.org/phylo/treebase/phylows/study/TB2:S22399?x-access-code=a01554a96d36b989fd14482c059b6b82&format=html).

## Results

### Identification of laccate *Ganoderma* collections

**Fig 1:**
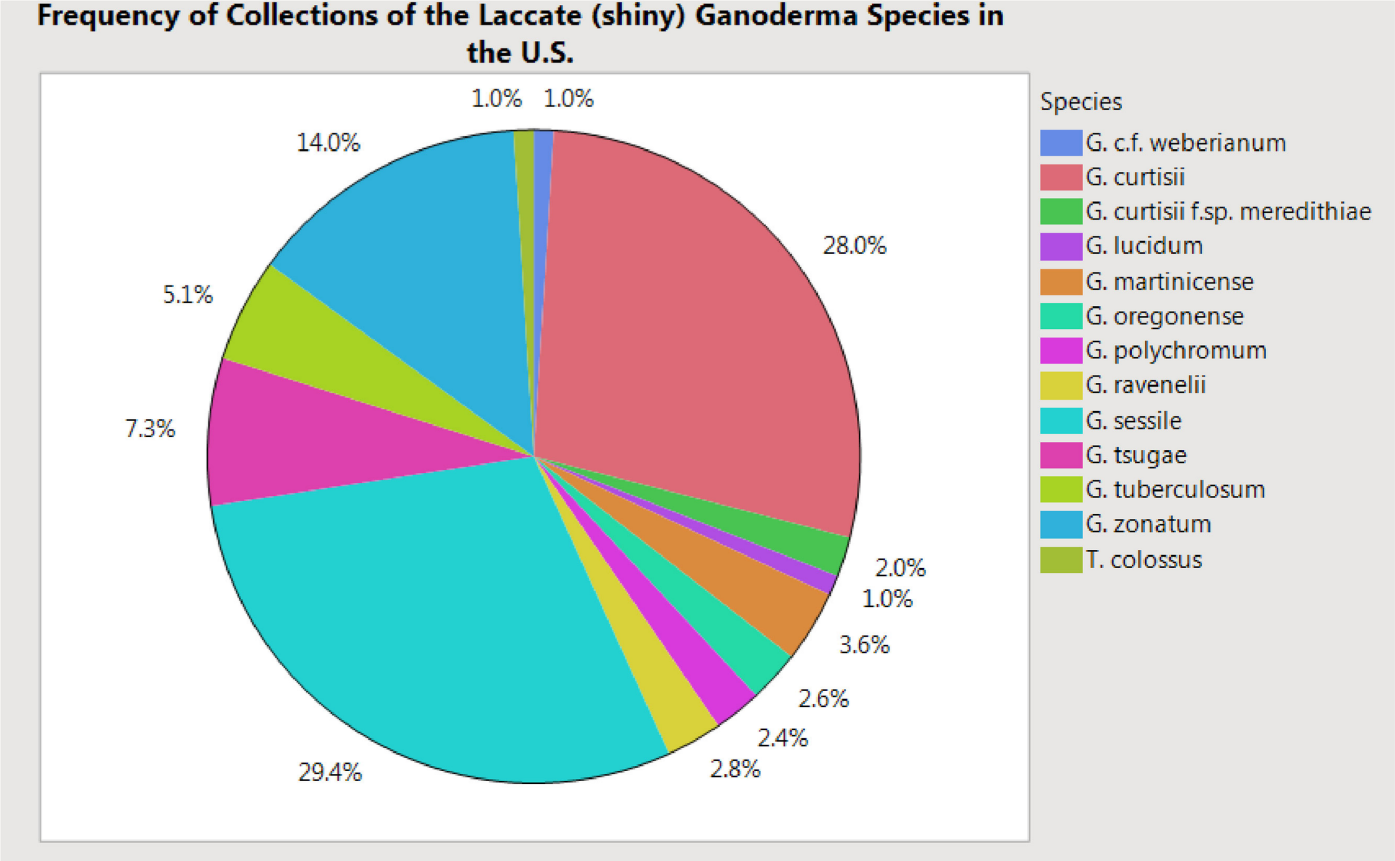
Frequency of taxa representing the laccate *Ganoderma* species collected in the United States. Percentages are representative of the total collections (n = 507). Species in the legend are represented in a clockwise order on the pie chart.

**Fig 2:**
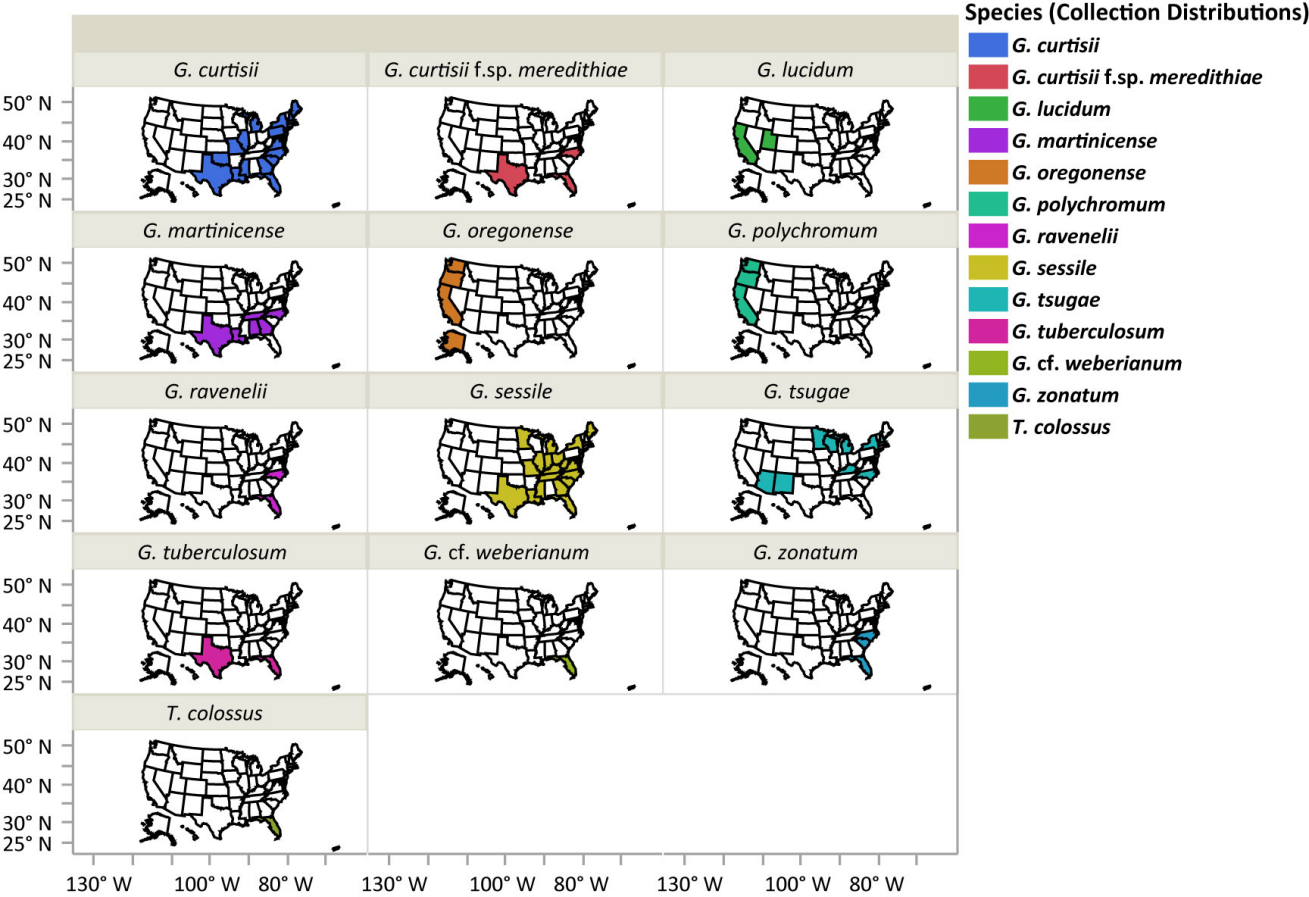
Distribution of collections of the laccate *Ganoderma* species studied. Species are shaded in different colors in each state where a collection of that species was made.

Five hundred and seven collections of laccate *Ganoderma* species (basidiomata and/or cultures) from our collections (n = 427) or from FLAS (n = 22) and NCSCLG (n = 58) mycological herbaria were studied (Fig 1). Collections originated from 34 U.S. states (Fig 2). Of the 22 specimens observed from FLAS, about half were collected and/or identified by Murrill, who was the taxonomic authority on all of these collections, including: *G*. *curtisii*, *G*. *oregonense*, *G*. *tuberculosum* and *G*. *zonatum*. Thirteen laccate taxa in the Ganodermataceae were identified in this U.S. survey, including *G*. *curtisii* (n = 142), *G*. *lucidum* (n = 5), *G*. *martinicense* (n = 18), *G*. *oregonense* (n = 13), *G*. *polychromum* (Copel.) Murrill (n = 12), *G*. *ravenelii* Steyaert (n = 14), *G*. *sessile* (n = 149), *G*. cf. *weberianum* (Bres. & Henn. ex. Sacc.) Steyaert (n = 5), *G*. *tsugae* (n = 37), *G*. *tuberculosum* (n = 26), *G*. *zonatum* (n = 71), *Tomophagus colossus* (n = 5) and *G*. *curtisii* f.sp. *meredithiae* (n = 10) f. sp. nov. Collections of *G*. *curtisii* f.sp. *meredithiae* represents collections formerly described as *G*. *meredithiae*. Here we synonymize this taxon with *G*. *curtisii* due to a lack of evidence to uphold the rank of species (see below). However, physiological differences, such as slow *in vitro* growth rate on MEA and an affinity to decay pine substrates, existed in collections of *G*. *meredithiae*, so the informal forma specialis designation, which is used as an informal taxonomic rank for physiological variants, is proposed here resulting with *G*. *curtisii* f.sp. *meredithiae*. The most commonly collected species in the eastern U.S. were *G*. *sessile* (29%), *G*. *curtisii* (28%) and *G*. *zonatum* (14%), while the more commonly encountered species in the western U.S. were *G*. *oregonense* (3%) and *G*. *polychromum* (2%) were the more commonly encountered species (Figs 1 and 2).

Although our collections were heavily biased towards the eastern U.S., there were some apparent geographic distribution limitations for some of the species (Fig 2). For example, *Tomophagus colossus* and *G*. cf. *weberianum* were only collected in subtropical south Florida. Similarly, *G*. *tuberculosum* was only collected in subtropical locations of south Florida and south Texas. Furthermore, collections of *G*. *oregonense* and *G*. *polychromum* were restricted to the western U.S. near each type locality, while collections of *G*. *curtisii*, *G*. *curtisii* f.sp. *meredithiae*, *G*. *martinicense*, *G*. *ravenelii*, *G*. *sessile*, *G*. *tsugae*, and *G*. *zonatum* were generally restricted to the eastern U.S. However, there were some anomalies with respect to geographic distributions. For example, *G*. *sessile* was collected twice in California and once in Utah, suggesting it may be more widespread or these were the results of introductions. Similarly, the collections of *G*. *lucidum* sensu stricto were from California and Utah, but were restricted to small geographic regions in anthropogenic environments in northern California and northern Utah. Furthermore, there were four collections of *G*. *tsugae* from the southwestern U.S., in northern New Mexico (UMNNM13 and UMNNM46) and northern Arizona (MS182AZ and UMNAZ9).

### Basidiomata morphology and host substrate affinities

Although there was some variation in basidiomata morphology within collections representing an individual species, there were generally several morphological features that were diagnostic. Diagnostic morphological features included I) stipitate vs. sessile fruiting body morphology, II) color of context tissue, III) contextual features (e.g melanized deposits), iv) presence/absence of chlamydospores. and v) shape and size of basidiospores.

Stipitate morphologies were designated based on having a stipe the same size or longer than the width of the pileus. Taxa that were generally stipitate, included: *G*. *curtisii*, *G*. *curtisii* f.sp. *meredithiae*, *G*. *lucidum*, and *G*. *ravenelii*. Sessile morphology of fruiting bodies were designated based on having basidiomata that lacked a stipe, or stipes, if present, were shorter than the width of the pileus (i.e. pseudostipitate). Species that generally produced sessile basidiomata included: *G*. *martinicense*, *G*. *oregonense*, *G*. *polychromum*, *G*. *sessile*, *G*. *tsugae*, *G*. *tuberculosum*, *G*. cf. *weberianum*, *G*. *zonatum*, and *Tomophagus colossus*. All collections of *G*. *tuberculosum* were truly sessile. Collections of *G*. *martinicense* were consistently centrally pseudostipitate, possessing a stipe that was less than half the width of the pileus. Similarly, collections of *G*. *oregonense*, *G*. *polychromum*, *G*. *sessile*, *G*. *tsugae G*. *zonatum*, *G*. cf. *weberianum* and *T*. *colossus* were rarely stipitate and occasionally pseudostipitate and generally the short stipe was produced laterally or off center.

**Fig 3:**
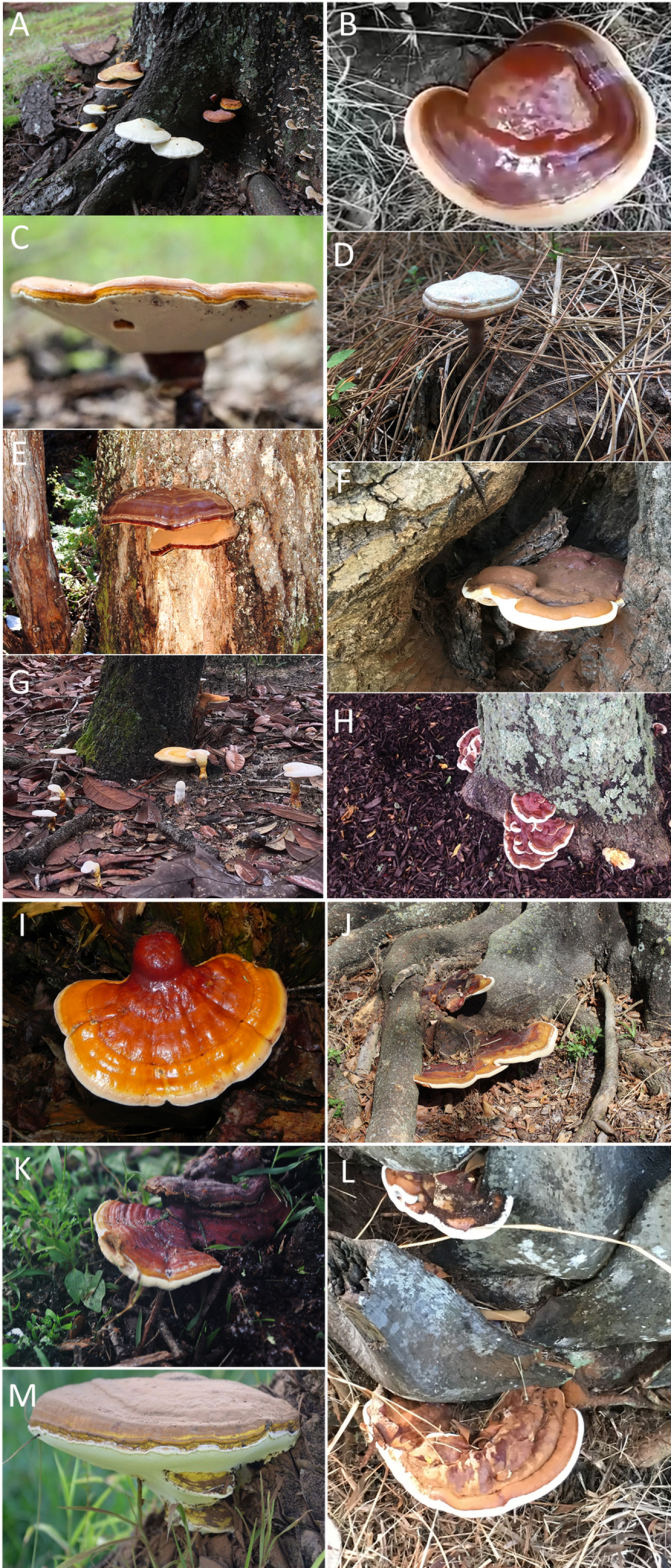
In situ photos of the laccate *Ganoderma* species in the United States. A) *G*. *curtisii* fruiting at the base of a dead oak tree (*Quercus* sp.) in Georgia (290GA), B) *G*. *lucidum* fruiting from near an oak (*Quercus* sp.) in California (*not in collection*) *(*photo credit: Shane Hanofee), C) *G*. *martinicense* fruiting from a southern red oak *(Quercus falcata*) in Georgia (230GA) (photo credit: Bill Sheehan), D) *G*. *curtisii* f. sp. *meredithiae* fruiting from slash pine roots in Florida (140FL), E) *G*. *oregonense* fruiting on white fir (*Abies concolor*) in California (no collection data) *(*photo credit: Arthur Grupe), F) *G*. *polychromum* fruiting on a pruning wound on a coast live oak (*Quercus agrifolia*) in California (331CA) (photo credit: Drew Zwart), G) *G*. *ravenelii* fruiting from the roots of an oak tree (*Quercus* sp.) in Florida (no collection data), H) *G*. *sessile* fruiting on the lower bole and root flare of honeylocust (*Gleditsia tricanthos*) in New York (276NY) (photo credit: Margery Daughtrey), I) *G*. *tsugae* fruiting on the trunk of eastern hemlock *(Tsuga canadensis*) in Wisconsin (342WI), J) *G*. *tuberculosum* fruiting on the root flare of pongam tree (*Pongamia pinnata*) in Florida (335FL), K) *G*. cf. *weberianum* near a recently removed live oak tree (*Quercus virginiana*) in Florida (261FL), L) *G*. *zonatum* fruiting on the trunk of an American oil palm (*Elaeis oleifera*) in Florida (283FL), and M) *Tomophagus colossus* fruiting on the cycad *Macrozamia moorei* in Florida (255FL) (photo credit: Michael Calonje).

**Fig 4:**
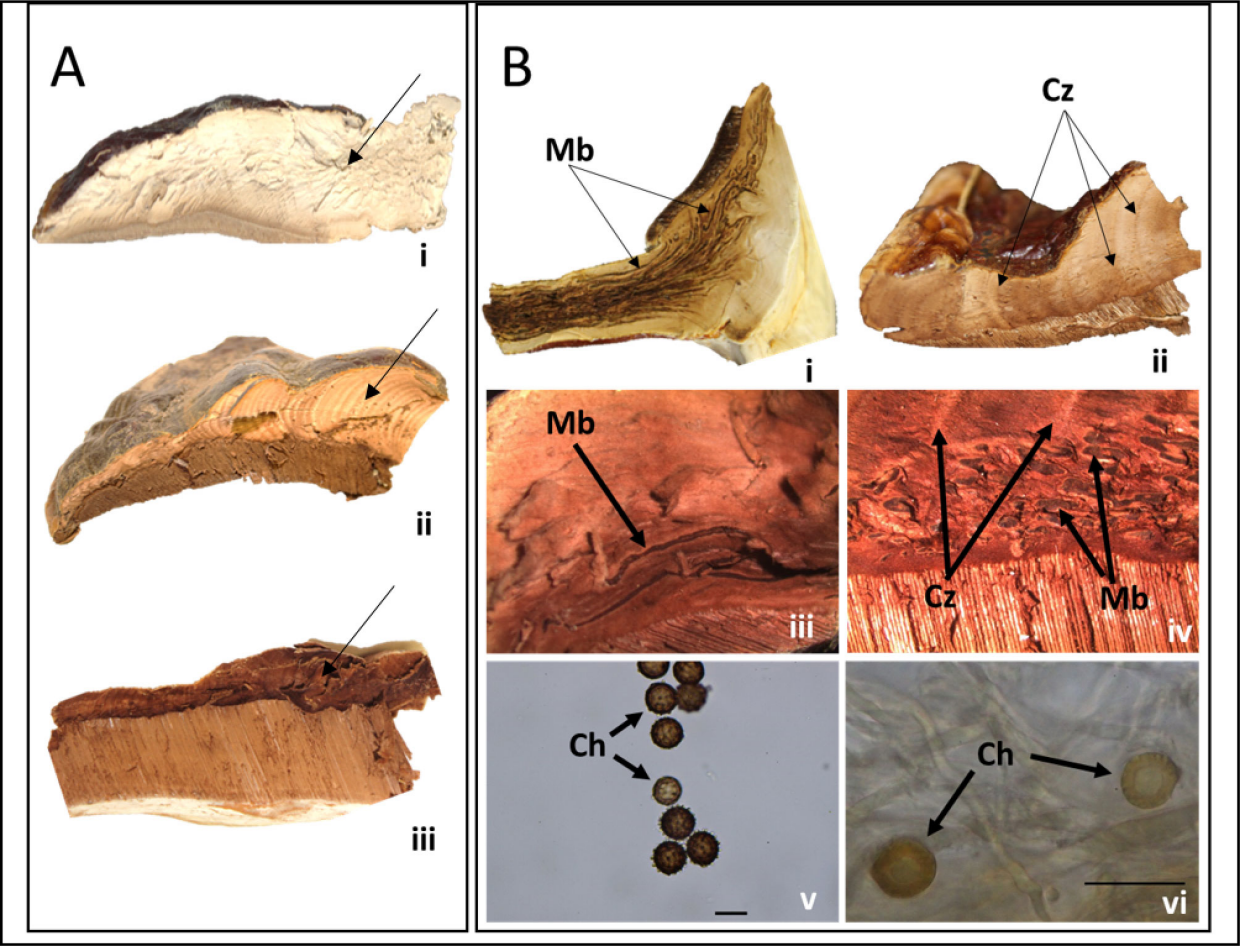
**Contextual colors (A) and features (B) of the laccate *Ganoderma* species of the U.S. (bars = 20 μm)** A) broad categories of the context tissue colors, where arrows point to context tissue; i) white context tissue (324WI, *G*. *tsugae*), ii) light buff to cream context tissue (112CA, *G*. *polychromum*), and iii) dark brown context tissue (265FL, *G*. *zonatum*). B) contextual features such as melanoid bands (“Mb”), concentric growth zones (“Cz”), and contextual chlamydospores (“Ch”); i) melanoid bands embedded in context tissue of pileus and stipe (158FL, *G*. *curtisii*), ii) concentric growth zones in context tissue of the pileus (171FL, *G*. *sessile*), iii) close-up (10x) of melanoid bands in the context tissue of the pileus (NCSCLG 1804, *G*. *curtisii* f.sp. *meredithiae*), iv) close-up (10x) of melanoid bands and concentric growth zones in context tissue of the pileus (NCSCLG 19006, *G*. *martinicense*), v) double walled, globose contextual chlamydospore (255FL, *T*. *colossus*), and vi) double walled contextual, globose chlamydospore with striated margin (FLAS F59210, *G*. cf. *weberianum*).

All thirteen taxa, had shiny or varnished (laccate) pilei that ranged in color from yellow to orange to reddish brown with variable coloration within a species (Fig 3). For example, pilei ranged in color from yellow-orange to reddish brown often with purple hues for collections of *G*. *curtisii*. Similarly, the pileus color of collections of *G*. *sessile* ranged from deep red to orangish-red. The color of the pileus was not diagnostic for most, although *T*. *colossus* was the only species that produced basidiomata that were distinctly shiny and mustard yellow. Similar to basidiomata color, pore shape and hymenium color were not always diagnostic. This is probably due to the age and maturity of the basidiomata as well as the environment where they were fruiting. However, the color of the context tissue was diagnostic. The context tissue is the inner flesh, or area between the pileus crust and initiation of the tubes. Generally, the color of the context tissue was grouped into three major categories: I) white, II) pinkish-buff to cinnamon-buff (i.e. light brown), or III) cinnamon brown (i.e. dark brown) (Fig 4A). All thirteen species could be grouped into one of these categories; *G*. *oregonense*, *G*. *tsugae*, and *T*. *colossus* had white context tissue, *G*. *curtisii*, *G*. *curtisii* f.sp. *meredithiae*, *G*. *lucidum* sensu stricto, *G*. *polychromum*, *G*. *ravenelii*, *G*. *sessile*, and *G*. cf. *weberianum* had buff to light brown, and *G*. *martinicense*, *G*. *tuberculosum*, and *G*. *zonatum* had dark cinnamon brown colored context tissues.

In addition to the color of the context tissue, certain context tissue features such as melanoid bands, concentric growth zones, and chlamydospores were diagnostic for many species (Fig 4B). For example, *G*. *curtisii*, *G*. *curtisii* f.sp. *meredithiae*, and *G*. *martinicense* had melanoid shiny deposits embedded in the context tissues. Similarly, *G*. *tuberculosum* often had resinous deposits in the context tissue, but were often lighter in color relative to the context tissue. Concentric growth zones (Fig 4B) were observed consistently in collections of *G*. *lucidum*, *G*. *martinicense*, *G*. *polychromum*, *G*. *sessile*, *G*. *tuberculosum*, *G*. cf. *weberianum*, and *G*. *zonatum*. Lastly, chlamydospores found in the context tissues were consistently observed in collections of *G*. cf. *weberianum* and *T*. *colossus*. The chlamydospores produced in fruiting body context tissue of *G*. cf. *weberianum* were hyaline or pigmented in 5% KOH, double-walled, ovate to globose, and on average were 17.1 (14.1–20.1) X 12.0 (9.6–14.1) μm. In addition the outer wall of chlamydospores of *G*. cf. *weberianum* were striate (Fig 4B-vi). The chlamydospores produced in fruiting body context tissue of *T*. *colossus* were pigmented, double-walled, globose, ornamented, and on average 16.1 (15.1–17.6) μm in diameter (Fig 4B-v). When grown on MEA, *G*. *martinicense*, *G*. *polychromum* and *G*. *sessile* also constitutively produced hyaline chlamydospores, but were rarely observed in the context tissue of basidiomata (Table 1).

**Table 1:** Morphological assessment of the laccate *Ganoderma* species present in the United States.

Taxon	Authority	Stipe			Context tissue^1^			Hymenium (pores/mm)	Chlamydospores^2^			Basidiospores^3^			
		presence	color	size	color/texture	concentric growth zones	resinous or melanoid deposits		present/absent	shape	size (length X width)	length	width	S.S.I.^4^	Q-ratio^5^
*G*. *curtisii*	(Berk.) Murrill 1902	almost always present	tawny to russett with occasional mauve pigments	typically, 1.5x the diameter of the cap	pink-buff to cinnamon buff/corky	absent	present	5–8	absent	-	-	10.6 (8.3–12.1) efg	6.4 (5.4–7.5) f	60.4 c	1.7 cd
*G*. *curtisii* f.sp. *meredithiae*	(Adask. & Gilb) (this paper)	almost always present	tawny to russett with occasional mauve pigments	typically, 1.5x the diameter of the cap	pink-buff to cinnamon buff/corky	absent	present	5–8	absent	-	-	10.8 (9.5–11.5) cd	6.8 (6.4–7.3 def)	62.6 bc	1.6 cd
*G*. *lucidum*	(Curtis) P. Karst 1881	almost always present	tawny to russett	long and eccentric; typically twice the diameter of the cap	pink-buff to cinnamon buff/corky	present	absent	4–5	absent	-	-	10.7 (8.2–12.1) f	7.1 (4.8–8.9) cd	66.2 ab	1.5 ef
*G*. *martinicense*	Welti & Court 2010	present	cinnamon brown to black	short and stubby; typically less than the diameter of the cap	cinnamon brown/felty to corky	present	present	5–6	present and abundant in culture	hyaline to pigmented ovate to spherical or irregularly shaped with hyphal appendages protruding out (culture)	17.1 (13.5–21.1) x 12.2 (9.2–17.3)	11.1 (9.0–13.6) def	6.9 (5.3–8.3) cde	62.6 abc	1.6 de
*G*. *oregonense*	Murrill 1908	occasionally present	tawny to cinnamon brown	short and stubby; typically less than the diameter of the cap	white/spongy to corky	absent	absent	3–4	absent	-	-	12.9 (11.6–14.9) b	8.0 (6.7–9.3) b	62.2 c	1.6 cd
*G*. *polychromum*	(Copel.) Murrill 1908	rarely present	tawny to russett	short and thin; typically less than the diameter of the cap if present	pink-buff to cinnamon-buff/corky	present	absent	4–5	present; rare in context and abundant in culture	elliptical to obpyriform to ovate, hyaline, smooth (culture)	14.8 (10.3–18.3) X 9.9 (11.9–7.0)	12.2 (10.8–13.2) cd	6.8 (6.0–7.4) cde	55.5 abc	1.8 cd
*G*. *ravenelii*	Steyaert 1980	almost always present	tawny to russett	typically, 1.5x the diameter of the cap	pink-buff to cinnamon-buff/corky	absent	absent	6–7	absent	-	-	11.2 (9.1–13.6) cde	5.2 (4.2–6.8) h	46.5 d	2.2 a
*G*. *sessile*	Murrill 1902	occasionally present	tawny to russett	short and thin; typically less than the diameter of the cap if present	pink-buff to cinnamon-buff/corky	present	rarely present	5–7	present; rare in context and abundant in culture	elliptical to obpyriform to ovate, hyaline, smooth (culture)	16.0 (12.0–26.0) X 11.0 (9.5–12.0)	11.4 (9.7–14.0) cd	6.6 (5.2–8.4) ef	58.1 c	1.7 c
*G*. *tsugae*	Murrill 1902	occasionally present	tawny to cinnamon brown	short and stubby; typically less than the diameter of the cap	white/spongy to corky	absent	absent	5–7	absent	-	-	9.9 (8.9–11.5) g	6.5 (5.2–7.7) ef	65.2 abc	1.5 def
*G*. *tuberculosum*	Murrill 1908	absent	-	-	cinnamon brown/felty to corky	present	present	4–7	absent	-	-	10.5 (9.2–12.0) fg	7.3 (6.2–8.6) c	69.3 a	1.4 f
*G*. cf. *weberianum*	(Bres. & Henn. ex Sacc.) Steyaert 1972	absent	-	-	pink-buff to cinnamon-buff/corky	present	absent	5–7	present; context tissue and culture	ovate to globular and striate and pigmented (context tissue); elliptical to obpyriform to ovate, hyaline, smooth (culture)	17.1 (14.1–20.1) X 12.0 (9.6–14.1)	8.4 (7.7–9.5) h	5.6 (4.7–7.3) gh	67.0 abc	1.5 ef
*G*. *zonatum*	Murrill 1902	rarely present	yellow ocher to russett	short and stubby; typically less than the diameter of the cap	cinnamon brown/felty	present	absent	4–6	absent	-	-	11.8 (10.3–13.7) c	5.9 (5.0–6.6) g	49.5 d	2.0 b
*T*. *colossus*	(Fr.) Murrill 1905	absent	-	-	white to light buff/spongy	absent	absent	3–4	present; abundant in context and culture	globular with abundant ornamentations pigmented (context tissue and culture)	16.1 (15.1–17.6)	16.1 (14.6–17.3) a	10.4 (9.5–11.3) a	64.8 abc	1.5 def

1. Sterile tissue inside fruiting body between the pileus crust and initiation of the tubes

2. Survival structures that could be produced in the fruiting body context tissue and/or in vitro culture

3. Basidiospore measurments ending in different letters are statistically different (P<0.05) with Tukey’s HSD means separation

4. The “S.S.I.” is the spore shape index, which is calculated by (width/length)*100, and is used to quantitatively describe the shape

5. The “Q-ratio” is a ratio of the length:width

The shape and size of basidiospores were diagnostic for some species. It is possible with additional measurements that do not include the inflated or truncated, hyaline apex of the basidiospores that nuanced differences in length could be elucidated as seen in Hennicke et al. (2016) [29]. However, all of our measurements included the entire spore length from base to truncated apex, which was similar and comparable to older *Ganoderma* literature [46]. For example, basidiospores of *T*. *colossus* were nearly one and one-half to twice as large as the other species. Although the basidiospores of *T*. *colossus* were much larger relative to the other species, the length to width ratio (Q-ratio) was similar (Q = 1.5). The basidiospore Q-ratio was distinctly larger for collections of *G*. *ravenelii* (Q = 2.2) and *G*. *zonatum* (Q = 2.0), because the spores were much more elongated than they were wide relative to the other taxa. Contrary to that, the spore shape index ((width/length)*100) of *G*. *tuberculosum* was 69.3, suggesting that 69.3% of the length can be explained by the width, which were considered broadly ovate, or “squatty”. Lastly the length of basidiospores was diagnostic for collections of *G*. cf. *weberianum*, which had basidiospores that were shorter than most (8.4(7.7–9.5) μm). Morphological descriptions are summarized in Table 1.

**Fig 5:**
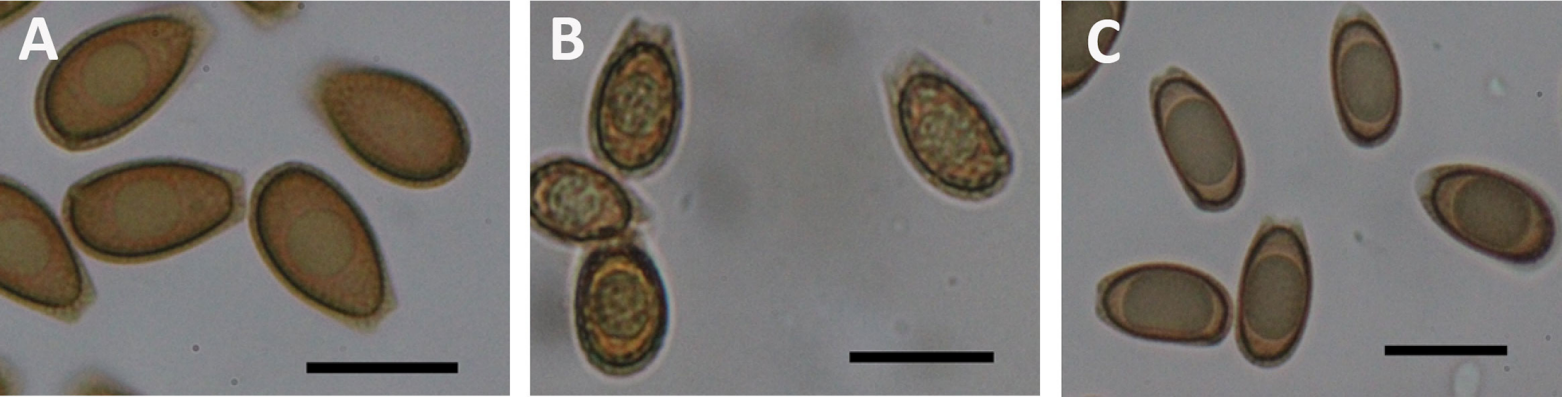
Examples of basidiospore morphology (bars = 10 μm). A) “smooth” (finely echinulated) from *G*. *sessile* (287SC), B) “rough” (coarsely echinulated) basidiospores from *G*. *curtisii* (158FL), and C) elongated, elliptical basidiospores of *G*. *zonatum* (265FL).

In addition to shape and size, the amount of echinulation of the basidiospores was diagnostic, and grouped into categories of “rough” or “smooth” as described previously [8, 12]. Collections of *G*. *sessile*, *G*. *polychromum* and *G*. cf. *weberianum* had “smooth” basidiospores due to fine echinulations protruding through the perisporium wall from the endosporium. The other taxa produced basidisopores that could be lumped into the “rough” basidiospore category, due to coarse echinulations protruding through the perisporium (outer, hyaline wall) from the endosporium (pigmented, inner portion of the spore) (Fig 5).

**Fig 6:**
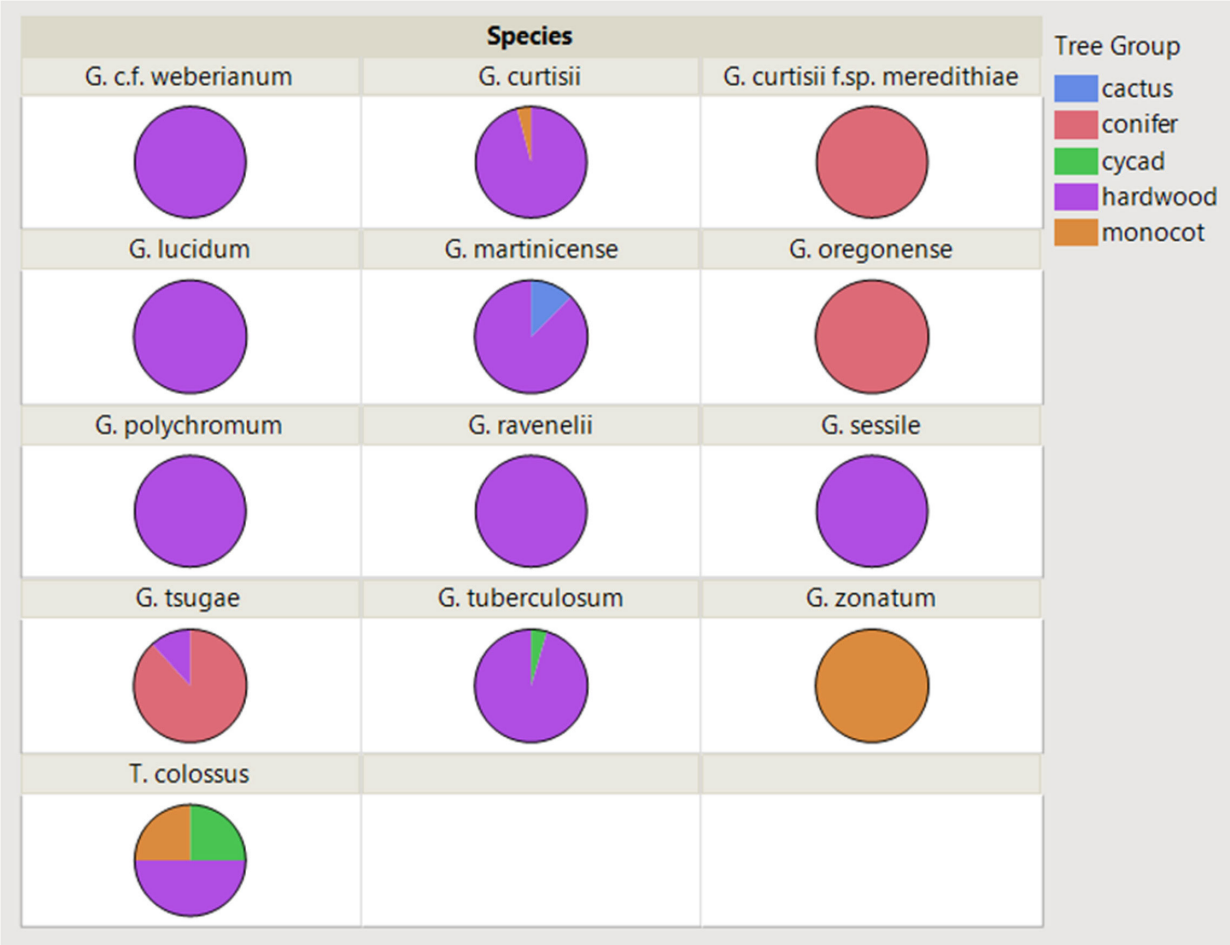
Host substrate affinities for the laccate (shiny) *Ganoderma* species collected in the U.S., where host substrate was known (n = 298).

Of the collections of laccate species of *Ganoderma* in the U.S. from this study, hosts were recorded for 298 collections. Of the known hosts/substrates for each collection 49 genera of plant species were identified across all collections, where the most frequent host genera were *Quercus* (37%), *Tsuga* (11%), *Sabal* (6%), and *Acer* (5%). The known host/substrate were categorized into the following broad categories: hardwood (68%), conifer (14%), monocot (17%), cycad (1%), and cactus (0.3%) (Fig 6). There were different affinities for certain groups of hosts across the different *Ganoderma* taxa. The following taxa were exclusively or predominately associated with hardwood host/substrate: *G*. *curtisii*, *G*. *martinicense*, *G*. *polychromum*, *G*. *ravenelii*, *G*. *sessile*, *G*. *tuberculosum*, and *G*. cf. *weberianum*. In addition, *G*. *curtisii* f.sp. *meredithiae*, *G*. *oregonense*, and *G*. *tsugae* were found fruiting predominately on conifers. Similarly, *G*. *zonatum* was only collected from monocots, mostly palm species, but three collections were from *Bambusa vulgaris*, a clumping bamboo. A species key of the laccate *Ganoderma* in the United States is presented using morphology, geography and host preference (Table 2).

**Table 2:** Key to the laccate *Ganoderma* species in the United States. Members of the laccate *Ganoderma* are have shiny or varnished pilei, and can be sessile, stipitate or pseudostipitate. The context tissue of the laccate *Ganoderma* species is corky to felty in texture, and generally white, cream to light buff, or cinnamon brown. Context tissue can have melanoid/resinous bands and/or concentric growth zones present or absent. Some taxa produce contextual chlamydospores that are double-walled, hyaline to pigmented, and ornamented or smooth. The basidiospores are double-walled, golden-brown in 5% KOH, echinulated and generally broadly ovoid to elliptical with a truncated apex at maturity. Ecologically these species are associated with a white rot type decay typically on hardwood, coniferous, or palm substrates. **Disclaimer:** This key is solely based on morphology, host preference and known geographic distributions of the laccate *Gaonderma* present in the U.S. based on this study.

1	Context tissue white when fresh	2
1	Context tissue not as above	4
2	Large basidiospores measuring on average 16.1 (14.6–17.3) x 10.4 (9.5–11.3) μm; fruiting body spongy, light-weight, shiny, and yellow	*T*. *colossus* (syn. = *G*. *colossus*)
2	Basidiospores smaller than above	3
3	Found on conifers in the Pacific Northwest, with basidiospores measuring on average 12.9 (11.6–14.9) x 8.0 (6.7–9.3) μm	*G*. *oregonense*
3	Found on conifers, predominately *Tsuga canadensis*, in the boreal hemlock forests of the eastern U.S. with basidiospores measuring on average 9.9 (8.9–11.5) x 6.5 (5.2–7.7) μm	*G*. *tsugae*
4	Context tissue cream to buff	5
4	Context tissue dark brown (cinnamon brown)	11
5	Shiny, melanoid deposits present in the context tissue	6
5	Melanoid bands absent, concentric growth zones sometimes present in the context tissue	7
6	Typically a laterally stipitate fruiting body, fruiting on hardwoods in the eastern U.S with basidiospores on average measuring 10.6 (8.3–12.1) x 6.4 (5.4–7.5), and growing somewhat rapidly (approximately 6 mm/day) on malt extract agar	*G*. *curtisii*
6	Typically a laterally stipitate fruiting body, fruiting on pines in the southeastern U.S with basidiospores on average measuring 10.8 (9.5–11.5) x 6.8 (6.4–7.3), and growing slowly (less than 3 mm/day) as a dikaryotic isolate on malt extract agar	*G*. *curtisii* f.sp. *meredithiae*
7	Typically sessile fruiting body morphology, or if stipe present, considered a pseudostipe, where the length of the stipe is less than the width of the pileus	8
7	Typically laterally stipitate fruiting body with no melanoid bands and concentric growth zones sometimes present in the context tissue	10
8	Found in association with hardwoods in the western United States, often with conspicuous concentric growth zones present in the context tissue	*G*. *polychromum*
8	Found in the eastern United States	9
9	Pigmented, double-walled globose to ovoid chlamydospores found in the context tissue, and restricted to tropical locations	*G*. cf. *weberianum*
9	Widely distributed East of the Rocky Mountains predominately associating with hardwood trees/substrates, basidiospores measuring on average 11.4 (9.7–14.0) x 6.6 (5.2–8.4) μm	*G*. *sessile*
10	Concentric growth zones absent from the context tissue, present in the southeastern U.S., and basidiospores measuring on average 11.2 (9.1–13.6) x 5.2 (4.2–6.8) μm	*G*. *ravenelii*
10	Concentric growth zones present in the context tissue, and restricted to isolated populations in northern Utah and northern California	*G*. *lucidum*
11	Basidiospores elongated measuring on average 11.8 (10.3–13.7) x 5.9 (5.0–6.0) μm, and associated with monocot trees/substrates, typically palms	*G*. *zonatum*
11	Not as above	12
12	Typically producing a central pseudostipe that is often dark red to black, associated with hardwood trees/substrates, concentric growth zones and melanoid deposits present in context tissue, and hymenium on average having 5–6 pores/mm	*G*. *martinicense*
12	Sessile fruiting body that is orange to red when active, and dark red when mature, concentric growth zones and shiny, resinous deposits present in the context, hymenium on average having 4–7 pores/mm, restricted to tropical locations, and basidiospores measuring on average 10.5 (9.2–12.0) x 7.3 (6.2–8.6) μm	*G*. *tuberculosum*

### Multilocus-based phylogeny of laccate *Ganoderma* species in the United States of America

In total, 522 sequences were generated in this study representing the thirteen species, and deposited into GenBank. These sequences represented 366 ITS (MG654066-MG654431), 60 *tef1* (MG754723-MG754782), 68 *rpb1* (MG754783-MG754850), and 27 *rpb2* (MG754851-MG754877) sequences (S1 Table). We were unable to obtain sequences from specimens that were older than five years. Representative sequences of each species were used in the phylogenetic analyses. In addition, selected voucher sequences were downloaded from GenBank (Table 3), and were selected as reference sequences, since they were used in a recent global phylogeny of members of the *G*. *lucidum* species complex [2, 28]. The only taxa that could not be validated with a voucher sequence or collections were *G*. *polychromum*, *G*. *ravenelii*, and *G*. cf. *weberianum* but these were clearly distinct genetically and generally matched the published taxonomic descriptions and type locations.

**Table 3:** Sample accessions, location, and GenBank Accession numbers for ITS, tef1α, rpb1, and rpb2 used in phylogenetic analysis.

Accession	Taxon	Location	GenBank Accession Numbers^1^			
			ITS	*tef1α*	*rpb1*	*rpb2*
WD2028	*Ganoderma boninense*	Japan	KJ143905	KJ143924	KJ143944	KJ143964
WD2085	*Ganoderma boninense*	Japan	KJ143906	KJ143925	KJ143945	KJ143965
102NC	*Ganoderma curtisii*	NC, USA	**MG654074**	**MG754727**	**—**	**MG754851**
223FL	*Ganoderma curtisii*	FL, USA	**MG654167**	**—**	**MG754785**	**MG754854**
238FL	*Ganoderma curtisii*	FL, USA	**MG654171**	**—**	**MG754786**	**—**
CBS100131	*Ganoderma curtisii*	NC, USA	JQ781848	KJ143926	KJ143946	KJ143966
CBS100132	*Ganoderma curtisii*	NC, USA	JQ781849	KJ143927	KJ143947	KJ143967
UMNFL28	*Ganoderma curtisii*	FL, USA	**MG654097**	**MG754728**	**MG754788**	**MG754856**
UMNFL6	*Ganoderma curtisii*	FL, USA	**MG654093**	**—**	**—**	**—**
UMNFL60	*Ganoderma curtisii*	FL, USA	**MG654105**	**MG754729**	**MG754789**	**—**
UMNGA1	*Ganoderma curtisii*	GA, USA	**MG654117**	**MG754731**	**MG754791**	**MG754857**
UMNNC3	*Ganoderma curtisii*	NC, USA	**MG654130**	**MG754732**	**MG754794**	**—**
124FL	Ganoderma curtisii f.sp. meredithiae	FL, USA	**MG654188**	**MG754734**	**MG754805**	**MG754861**
UMNFL50	Ganoderma curtisii f.sp. meredithiae	FL, USA	**MG654103**	**MG754735**	**MG754806**	**MG754862**
UMNFL64	Ganoderma curtisii f.sp. meredithiae	FL, USA	**MG654106**	**—**	**MG754807**	**MG754863**
Wei5491	*Ganoderma flexipes*	Hainan, China	JQ781850	–	–	KJ143968
Wei5494	*Ganoderma flexipes*	Hainan, China	JN383979	—	—	—
Cui9166	*Ganoderma lingzhi*	Shandong, China	KJ143907	JX029974	JX029982	JX029978
Dai12479	*Ganoderma lingzhi*	Anhui, China	JQ781864	JX029975	JX029983	JX029979
Cui9207	*Ganoderma lucidum*	Yunan, China	KJ143910	KJ143928	KJ143949	KJ143970
K175217	*Ganoderma lucidum*	United Kingdom	KJ143911	KJ143929	KJ143950	KJ143971
MS183CA	*Ganoderma lucidum*	CA, USA	**MG911000**	**MG754723**	**—**	**—**
MT26/10	*Ganoderma lucidum*	Czech Republic	KJ143912	KJ143930	KJ143951	—
Rivoire4195	*Ganoderma lucidum*	France	KJ143909	—	KJ143948	KJ143969
UMNUT1	*Ganoderma lucidum*	UT, USA	**MG654070**	**MG754725**	**MG754799**	**—**
UMNUT7	*Ganoderma lucidum*	UT, USA	**MG654071**	**MG754726**	**MG754800**	**—**
UMNUT8	*Ganoderma lucidum*	UT, US	**MG654072**	**—**	**—**	**—**
UMNUT9	*Ganoderma lucidum*	UT, US	**MG654073**	**—**	**—**	**—**
231NC	*Ganoderma martinicense*	NC, USA	**MG654182**	**MG754736**	**MG754801**	**—**
246TX	*Ganoderma martinicense*	TX, USA	**MG654185**	**MG754737**	**MG754802**	**MG754858**
LIPSW-Mart08-44	*Ganoderma martinicense*	Martinique	KF963257	—	—	—
LIPSW-Mart08-55	*Ganoderma martinicense*	Martinique	KF963256	—	—	—
UMNAL2	*Ganoderma martinicense*	AL, USA	**MG654176**	**—**	**—**	**—**
UMNSC7	*Ganoderma martinicense*	SC, USA	**MG654177**	**—**	**—**	**MG754859**
UMNTN1	*Ganoderma martinicense*	TN, USA	**MG654178**	**MG754738**	**MG754803**	**MG754860**
UMNTX3	*Ganoderma martinicense*	TX, USA	**—**	**MG754739**	**MG754804**	**—**
CWN04670	*Ganoderma multipileum*	Tawian, China	KJ143913	KJ143931	KJ143952	KJ143972
Dai9447	*Ganoderma multipileum*	Hainan, China	KJ143914	KJ143932	KJ143953	KJ143973
CBS265.88	*Ganoderma oregonense*	OR, USA	JQ781875	KJ143933	KJ143954	KJ143974
CBS266.88	*Ganoderma oregonense*	WA, USA	JQ781876	—	KJ143955	KJ143975
UMNAK1	*Ganoderma oregonense*	AK, USA	**MG654190**	**MG754740**	**MG754808**	**—**
UMNOR1	*Ganoderma oregonense*	OR, USA	**MG654194**	**MG754741**	**MG754809**	**—**
330OR	*Ganoderma polychromum*	OR, USA	**MG654196**	**MG754742**	**—**	**—**
BJ280CA	*Ganoderma polychromum*	CA, USA	**MG910492**	**—**	**—**	**—**
BJ316CA	*Ganoderma polychromum*	CA, USA	**MG910493**	**—**	**—**	**—**
MS343OR	*Ganoderma polychromum*	OR, USA	**MG654197**	**MG754743**	**—**	**—**
UMNOR3	*Ganoderma polychromum*	OR, USA	**MG654204**	**MG754744**	**MG754810**	**—**
MS187FL	*Ganoderma ravenelii*	FL, USA	**MG654211**	**MG754745**	**MG754813**	**MG754865**
UMNFL187	*Ganoderma ravenelii*	FL, USA	**—**	**—**	**MG754814**	**—**
UMNFL188	*Ganoderma ravenelii*	FL, USA	**—**	**MG754746**	**MG754815**	**—**
CBS194.76	*Ganoderma resinaceum*	Netherlands	KJ143916	KJ143934	KJ143956	
Rivoire4150	*Ganoderma resinaceum*	France	KJ143915	—	KJ143957	—
103SC	*Ganoderma sessile*	SC, USA	**MG654304**	**—**	**—**	**—**
111TX	*Ganoderma sessile*	TX, USA	**MG654306**	**MG754747**	**MG754816**	**MG754866**
113FL	*Ganoderma sessile*	FL, USA	**MG654307**	**MG754748**	**—**	**MG754867**
117TX	*Ganoderma sessile*	TX, USA	**MG654309**	**MG754749**	**MG754817**	**MG754868**
165MO	*Ganoderma sessile*	MO, USA	**MG654312**	**—**	**MG754818**	**—**
171FL	*Ganoderma sessile*	FL, USA	**MG654316**	**—**	**MG754819**	**—**
228DC	*Ganoderma sessile*	DC, USA	**MG654319**	**MG754750**	**MG754820**	**MG754869**
JV1209/27	*Ganoderma sessile*	AZ, USA	KF605630	KJ143937	KJ143959	KJ143976
NY00985711	*Ganoderma sessile*	NY, US	KJ143918	—	—	—
UMNCA5	*Ganoderma sessile*	CA, USA	**MG910998**	**—**	**—**	**—**
UMNFL10	*Ganoderma sessile*	FL, USA	**MG654227**	**MG754753**	**MG754821**	**—**
UMNFL125	*Ganoderma sessile*	FL, USA	**MG654239**	**MG754755**	**MG754825**	**—**
UMNFL19	*Ganoderma sessile*	FL, USA	**MG654230**	**MG754754**	**MG754822**	**—**
UMNKY1	*Ganoderma sessile*	KY, USA	**MG654257**	**MG754756**	**MG754827**	**—**
UMNMI22	*Ganoderma sessile*	MI, USA	**MG654269**	**MG754757**	**MG754828**	**—**
UMNMI24	*Ganoderma sessile*	MI, USA	**MG654271**	**MG754758**	**MG754829**	**—**
UMNNY14	*Ganoderma sessile*	NY, US	**MG654294**	**—**	**MG754830**	**—**
UMNOH4	*Ganoderma sessile*	OH, USA	**MG654298**	**MG754759**	**MG754831**	**—**
UMNWV1	*Ganoderma sessile*	WV, US	**MG654302**	**—**	**—**	**—**
Cui7691	*Ganoderma sichuanense*	Guangdong, China	JQ781878	—	—	—
HMAS42798 (Holytype)	*Ganoderma sichuanense*	Sichuan, China	JQ781877	—	—	—
Dai9724	*Ganoderma tropicum*	Hainan, China	JQ781879	—	—	—
Yuan3490	*Ganoderma tropicum*	Yunnan, China	JQ781880	KJ143938	—	—
Dai1275b	*Ganoderma tsugae*	CT, USA	KJ143919	KJ143939	KJ143960	KJ143977
Dai12760	*Ganoderma tsugae*	CT, USA	KJ143920	KJ143940	KJ143961	KJ143978
MS182AZ	*Ganoderma tsugae*	AZ, USA	**MG910999**			**MG754864**
UMNMI20	*Ganoderma tsugae*	MI, USA	**MG654324**	**MG754764**	**MG754836**	**—**
UMNMI30	*Ganoderma tsugae*	MI, USA	**MG654326**	**MH025362**	**MG754837**	**MG754871**
UMNNC4	*Ganoderma tsugae*	NC, USA	**MG654329**	**MG754765**	**MG754838**	**MG754872**
LIPSW-Mart08-45	*Ganoderma tuberculosum*	Martinique	KF96325	—	—	—
PLM684	*Ganoderma tuberculosum*	FL, USA	**MG654369**	**MG754769**	**—**	**—**
UMNFL160	*Ganoderma tuberculosum*	FL, USA	**MG654364**	**—**	**MG754840**	**—**
UMNFL82	*Ganoderma tuberculosum*	FL, USA	**—**	**MG754770**	**—**	**MG754874**
UMNFL100	*Ganoderma* cf. *weberianum*	FL, USA	**MG654373**	**MG754762**	**MG754834**	**—**
UMNFL32	*Ganoderma* cf. *weberianum*	FL, USA	**MG654372**	**MG754761**	**MG754833**	**—**
123FL	*Ganoderma zonatum*	FL, USA	**MG654416**	**MG754774**	**MG754841**	**—**
179NC	*Ganoderma zonatum*	NC, USA	**MG654417**	**MG754775**	**MG754842**	**MG754875**
FL-02	*Ganoderma zonatum*	FL, USA	KJ143921	KJ143941	KJ143962	KJ143979
GAN11	*Ganoderma zonatum*	FL, USA	**—**	**MG754776**	**—**	MG754876
UMNFL105	*Ganoderma zonatum*	FL, USA	**MG654408**	**MG754780**	**MG754847**	—
UMNFL137	*Ganoderma zonatum*	FL, USA	**MG654413**	**MG754781**	**MG754848**	—
UMNFL16	*Ganoderma zonatum*	FL, USA	**MG654381**	**MG754777**	**MG754843**	—
UMNFL85	*Ganoderma zonatum*	FL, USA	**MG654402**	**MG754778**	**MG754844**	**MG754877**
UMNFL89	*Ganoderma zonatum*	FL, USA	**MG654403**	**MG754779**	**MG754845**	**—**
UMNSC4	*Ganoderma zonatum*	SC, USA	**MG654415**	**MG754782**	**MG754849**	**—**
TC-02	*Tomophagus colossus*	Vietnam	**KJ143923**	**KJ143943**	**KJ143963**	**—**
UMNFL110	*Tomophagus colossus*	FL, USA	**MG654429**	** **	**MG754850**	—

1. Accession numbers in boldface were generated from this study

**Fig 7:**
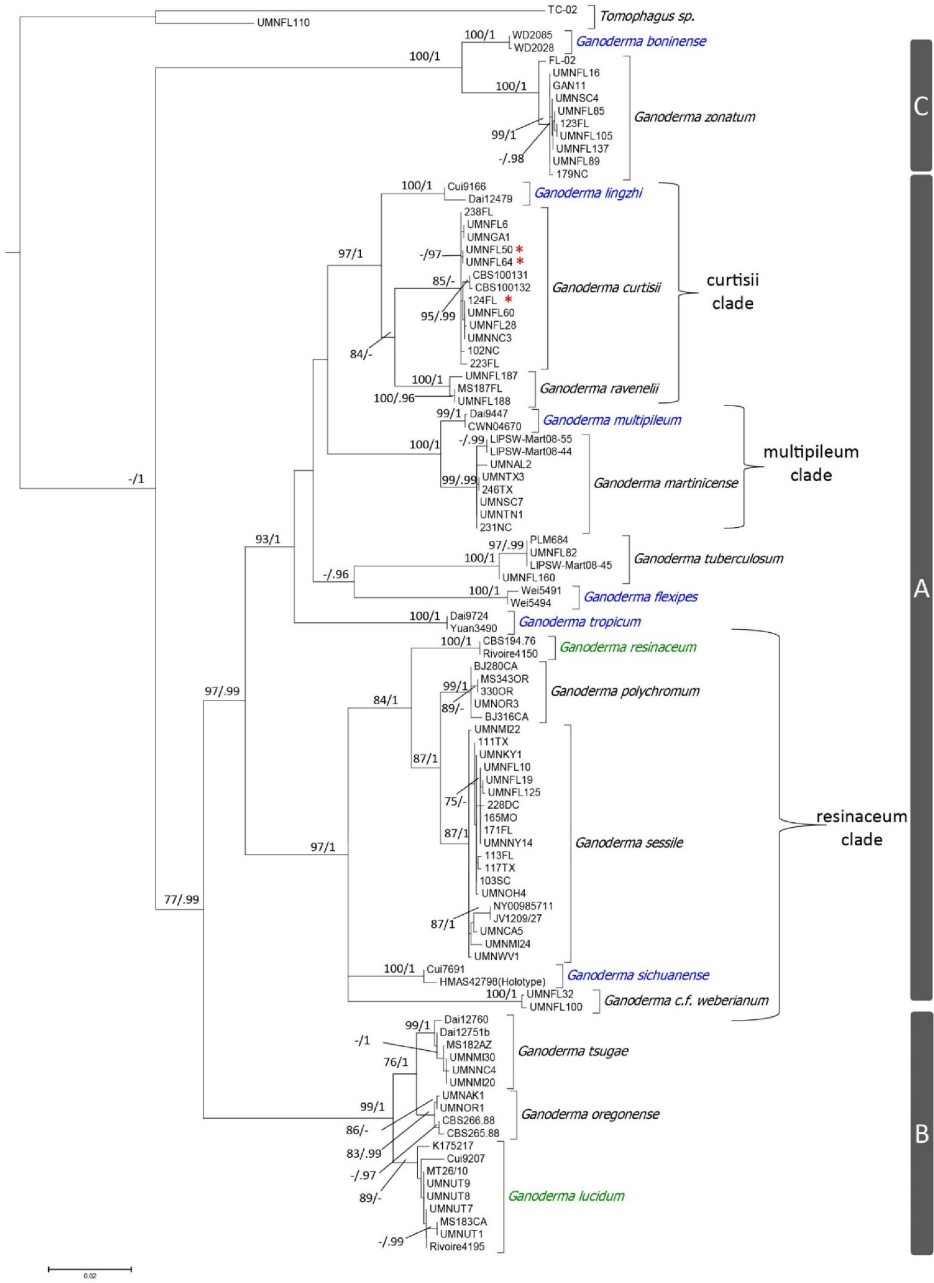
Tree topology derived from RAxML analysis of a multilocus alignment (ITS + *tef1α* + *rpb1* + *rpb2*) with 2470 characters under a GTR model with 1000 bootstrap replicates. Statistical values shown are ML-bootstrap values above 75%, and the second value is the posterior probability (PP) where values above 95% are shown. Species native to Asia are in blue font, species native to Europe are in green font, and species native to North American are in black font. Red asterisks indicate *G*. *curtisii* f.sp. *meredithiae*. There are three major clades (A, B, and C), and subclades with names derived from the taxon that was described first.

The phylogenetic analyses of the concatenated sequences with RAxML and Mr. Bayes yielded similar topologies, so the tree topology derived from the RAxML analysis was presented (Fig 7). Eighteen, well supported (ML-BS > 75%, and PP >95) terminal clades were resolved in the multilocus phylogeny using four loci representing 97 collections of the laccate *Ganoderma* species with *Tomophagus* species used as the outgroup (Fig 7). These 18 core *Ganoderma* terminal clades represented the following 18 taxa *G*. *boninense* Pat. 1889, *G*. *curtisii*, *G*. *flexipes* Pat. 1907, *G*. *lingzhi* Sheng H. Wu, Y. Cao & Y.C. Dai 2012, *G*. *lucidum*, *G*. *martinicense*, *G*. *curtisii* f.sp. *meredithiae* f.sp. nov., *G*. *multipileum*, *G*. *oregonense*, *G*. *polychromum*, *G*. *ravenelii*, *G*. *resinaceum*, *G*. *sessile*, *G*. *sichuanense* J.D. Zhao & X.Q. Zhang 1983, *G*. *tropicum* (Jungh.) Bres. 1910, *G*. *tsugae*, *G*. *tuberculosum*, *G*. cf. *weberianum*, and *G*. *zonatum*. Collections we labeled as *G*. *meredithiae*, based on the original species description, were conspecific with collections of *G*. *curtisii*, but the other 18 taxa represented well supported phylogenetic species. The 18 species in the presented phylogeny were broken into three highly supported subclades (Fig 7). The eighteen species represented a monophyletic group with high Bayesian posterior probability, but not with maximum likelihood. There did not appear to be clustering of taxa based on geographic location, but it appears there could be more genetic diversity and taxa in tropical and subtropical locations [21]. Clade labels used in Zhou et al. [2] were used in this phylogeny for consistency between studies.

Clade A was the most diverse clade, and contained thirteen of the eighteen species (Fig 7). Seven of thirteen were present in the United States. Although Clade A was highly supported as a major group separate from Clades B and C, there were also three well supported crown clades within this major clade. These clades were designated with the name based on the specific epithet of the taxon that was first described within the given crown clade. The “curtisii subclade” was highly supported, and included the North American taxa *G*. *curtisii*, and *G*. *ravenelii*, as well as the Asian taxon *G*. *lingzhi*. The “multipileum subclade” was highly supported, and included the Asian taxon *G*. *multipileum* and North American taxon *G*. *martinicense*. The “resinaceum subclade” was highly supported and included the European taxon *G*. *resinaceum*, the Asian taxon *G*. *sichuanense*, and the North American taxa *G*. *polychromum*, *G*. *sessile*, and *G*. cf. *weberianum*.

Clade B consisted of three well supported lineages including the European taxon *G*. *lucidum* sensu stricto, and the North American taxa *G*. *oregonense* and *G*. *tsugae*. Clade C consisted of two well supported lineages with the Asian taxon *G*. *boninense* and the North American *G*. *zonatum*. Lastly, *Tomophagus colossus* (UMNFL110) and *T*. *cattienensis* X.T. Le & Moncalvo [47] (TC-02) were used as outgroups because *Tomophagus* is a closely related genus in the Ganodermataceae.

### Determining the optimal DNA barcode locus for *Ganoderma* species

The RAxML analyses of the four individual loci independently and concatenation of four loci resulted in the following cumulative bootstrap scores as described previously: ITS (761) with eight terminal clades, *tef1α* (1070) with twelve terminal clades, *rpb1* (955) with eleven terminal clades, *rpb2* (564) with eight terminal clades, and ITS + *tef1α* + *rpb1* + *rpb2* (1141) with twelve terminal clades. Samples of *G*. *meredithiae* were not resolved with any individual locus or multilocus phylogenetic analysis. Based on these data, *tef1α* and *rpb1* resolve the laccate *Ganoderma* species better than the primary fungal barcode region ITS. Although there were much fewer samples with *rpb2* sequences, we suspect that *rpb2* would show similar or better resolution compared to *tef1α* and *rpb1* based on previous work from Matheny et al. [40]. Furthermore, some terminal clades for *tef1α*, *rpb1*, and *rpb2* had only one individual sample representative, so statistical values could not be assigned (Table 4).

**Table 4:** Comparisons of terminal clades and associated bootstrap scores for the laccate Ganoderma taxa present in the United States using RAxML analysis with 1,000 bootstrap replications for ITS, tef1α, rpb1, rpb2, and ITS+*tef1α*+ *rpb1*+*rpb2*.

	Phylogenetic Analyses Terminal Clade RAxML Bootstrap Scores				
Taxon	ITS	*tef1α*	*rpb1*	*rpb2*	ITS + *tef1α* + *rpb1* + *rpb2*
*G*. *curtisii*	89^1^	100	100	100	85
*G*. *curtisii* f.sp. *meredithiae*	NR^2^	NR	NR	NR	NR
*G*. *lucidum*	NR	98	91	86	89
*G*. *martinicense*	93	R*^3^	100	100	99
*G*. *oregonense*	NR	98	78	NR	83
*G*. *polychromum*	97	90	R*	NT	99
*G*. *ravenelii*	85	100	100	NR*	100
*G*. *sessile*	48	100	57	100	87
*G*. *tsugae*	NR	93	100	81	99
*G*. *tuberculosum*	100	R*	87	NR*	100
*G*. cf. *weberianum*	97	100	100	NT	100
*G*. *zonatum*	100	91	99	97	100
*T*. *colossus*	100	100	100	NT	100
**TOTAL SCORE**^**4**^	**761**	**970**	**955**	**564**	**1141**
**NUMBER OF TERMINAL CLADES**	**8**	**12**	**11**	**8**	**12**

1. Numbers represent maximum likelihood bootstrap scores of terminal clades for each taxon from a RAxML analysis using rapid bootstrapping, a GTR evolutionary model, and 1000 boostrap replications

2. “NR” represents non-resolved terminal clades

3. “R*” represents clades where statistical value could not be computed, but lineage resolved

4. Summation of bootstrap support values for well-supported terminal clades

## Discussion

The results of this study resolve problems and will help reduce confusion that is associated with the taxonomy of the *G*. *lucidum* species complex in the United States [1]. The comprehensive survey of laccate species of *Ganoderma* in the U.S. and the multilocus phylogeny helps resolve the species that were historically combined together as *G*. *lucidum* sensu lato. In addition to the five taxa presented in the treatise “North American Polypores” [1], eight more taxa are recognized within the United States, and include *G*. *curtisii*, *G*. *curtisii* f.sp. *meredithiae*, *G*. *martinicense*, *G*. *polychromum*, *G*. *ravenelii*, *G*. *sessile*, *G*. *tuberculosum*, and *G*. cf. *weberianum*. Furthermore, *G*. *colossum*, which was recognized in “North American Polypores” [1] has recently been placed back in the genus *Tomophagus* based on molecular data [22]. Lastly, the European taxon *G*. *lucidum* sensu stricto was found only in disturbed habitats in geographically restricted areas of northern Utah and northern California, suggesting they are likely isolated introduction events of a non-native species.

On a global scale, we found 19 well-supported terminal clades were formed with the multilocus phylogeny, including twelve species in the Ganodermataceae present in the U.S., six from Asia, and two from Europe. These 19 clades represented three highly supported clades in the genus *Ganoderma* and one highly supported clade in the genus *Tomophagus*, which was used as the outgroup. Within the core *Ganoderma* clades (A, B and C), Clade C that was comprised of *G*. *boninense* and *G*. *zonatum* was found to be the most basal clade. Both *G*. *boninense* and *G*. *zonatum* decay palms, which are an ancient lineage of plants [48]. We hypothesize that this relationship could explain their basal position on our phylogenetic tree, although detailed phylogenetic dating analyses are needed to address this possibility. Clade B, comprised of *G*. *lucidum* sensu stricto, *G*. *oregonense*, and *G*. *tsugae*, which includes temperate species, which suggests members of this clade share a common ancestor that was adapted for temperate climates. Finally, Clade A is the most derived and diverse clade of the laccate *Ganoderma* species with mostly subtropical to tropical species, including a few taxa that were geographically widespread.

Clade A was broken up into the three well-supported crown clades, the “curtisii subclade”, the “multipileum subclade”, and the “resinaceum subclade”. The “curtisii subclade” contained the three distinct species *G*. *curtisii*, *G*. *lingzhi*, and *G*. *ravenelii*. *Ganoderma lingzhi* is an Asian species that historically was also considered *G*. *lucidum* sensu lato, and is one of the most widely cultivated species for medicinal use [29, 49]. Although there is little information on the medicinal benefits of North American *Ganoderma* taxa, it is likely, given the phylogenetic placement of *G*. *curtisii* and *G*. *ravenelii*, that these species would produce similar pharmaceutically relevant compounds. All three of these species produce basidiomata that are generally laterally stipitate. *Ganoderma curtisii* is widespread throughout the eastern United States, and possesses melanoid deposits in the context tissue of the pileus and stipe. The basidiomata of *G*. *meredithiae* are morphologically indistinguishable from collections of *G*. *curtisii*, and based on the presented phylogeny constructed with four loci (ITS, *tef1α*, *rpb1*, and *rpb2*), *G*. *meredithiae* is considered to be conspecific with *G*. *curtisii*. Originally, *G*. *meredithiae* was circumscribed as a novel species based on the affinity to colonize and decay pines and slow *in vitro* growth rate on MEA [20]. Similar physiological differences between collections of *G*. *meredithiae* and *G*. *curtisii* were found in an *in vitro* growth study with our collections[50]. Furthermore, in another study, when isolates of *G*. *meredithiae* were grown on artificial media amended with pine water-soluble sapwood extracts, the linear growth was enhanced relative to the malt extract agar control. In contrast, *G*. *curtisii* was negatively affected by media amended with pine sapwood extracts relative to the MEA control [50]. Based on these physiological differences, we feel it necessary to discuss the two taxa separately, and use the *forma specialis meredithiae* designation for this physiological variant of *G*. *curtisii*. Based on the taxonomic code for fungi, *forma specialis* are used as informal classification to distinguish physiological variants of a given parasite species [51].

*Ganoderma ravenelii* was originally described by Steyaert [12], and was considered highly similar to *G*. *curtisii*. Type collections were from Florida, and additional specimens from South Carolina were also used to describe this species [12]. This species was originally differentiated from collections of *G*. *curtisii* by having more elongated basidiospores and lacking melanoid bands within the context tissues of the pileus and stipe [12]. Similarly, our collections studied from Florida and North Carolina lacked melanoid bands in the context tissue, and consistently had basidiospores that were more elongated than the basidiospores of *G*. *curtisii*. The average length to width ratio (Q ratio) of basidiospores of *G*. *ravenelii* was 2.2, while the Q ratio of basidiospores of *G*. *curtisii* collections was 1.7. Furthermore, this species was differentiated with high statistical support from collections of *G*. *curtisii* with all of the loci (ITS, *tef1α*, *rpb1* and *rpb2*) individually. Interestingly, this species has an overlapping geographic distribution with *G*. *curtisii*, and more studies focusing on the ecology of this species are needed to understand if there are functional differences between the two species.

There were only two species included in this phylogeny from the “multipileum subclade”, which were *G*. *multipileum* and *G*. *martinicense*. This clade is sister to the “curtisii subclade”. *Ganoderma multipileum* is an Asian species that was historically labeled as *G*. *lucidum* sensu lato in tropical Asian locations [28]. The species *G*. *martinicense* was described from Martinique, and was suspected to be the vicariant relative of *G*. *multipileum* [28]. Our phylogeny placed these geographically distant species as sister taxa, which corroborates the hypothesis of vicariant evolution. *Ganoderma martinicense* is diagnosed by having dark brown context tissue, a centrally produced pseudostipe that is dark red to black, and melanoid bands as well as concentric growth zones in the context tissue. This survey reports *G*. *martinicense* from Alabama, Georgia, Louisiana, Mississippi, North Carolina, South Carolina, Tennessee, and Texas (U.S.). We suspect that *G*. *martinicense* has an even wider geographic distribution throughout the southeastern U.S. and the Caribbean. More surveys in North, Central and South America will help elucidate the geographic distribution of this species.

The “resinaceum subclade” is a diverse clade that includes species that generally produced sessile, and occasionally pseudostipitate fruiting bodies. Furthermore, all of the species within this subclade constitutively and vegetatively-produce double-walled chlamydospores with a smooth surface *in vitro* on MEA, which is an apomorphic characteristic [22]. The species *G*. *resinaceum*, the subclade’s name sake, is a European species that was historically thought to be a synonym for *G*. *sessile* and *G*. *polychromum* and the North American *G*. *lucidum* sensu lato [12, 17, 52]. Based on our phylogeny, European members of the “resinaceum subclade” represent a distinct lineage from the North American *G*. *sessile* and *G*. *polychromum* lineages, which are sister to one another. Previous research [53] suggests laboratory biological compatibility of monokaryotic European and North American sister species is not uncommon, and occasionally can result in dikaryotic isolates with reduced vigor, such as observed in intersterility groups of members of the European and North American *Armillaria mellea* species complex [53]. Furthermore, gene flow is naturally improbable with taxa that are separated by geographic barriers such as oceans and mountain ranges or taxa that do not have long distance dispersal methods [18]. Therefore, taxa that are in the process of allopatric speciation may not be intersterile because there is no mechanism that has evolved to prevent outcrossing [18].

There are three distinct North American linages representing three unique species within the resinaeum subclade, which include *G*. *polychromum*, *G*. *sessile*, and *G*. cf. *weberianum*. *Ganoderma polychromum*, (syn. = *Polyporus polychromus* Copel.), was described from California and Nevada, and the zonate context tissue and affinity for angiosperms were considered diagnostic features [7, 12]. This species, like many of the others from the U.S., has long been mislabeled as *G*. *lucidum* sensu lato. *Ganoderma polychromum* is the sister taxon to *G*. *sessile*, a species widespread in the eastern U.S.

*Ganoderma sessile* is the most taxonomically controversial of all laccate *Ganoderma* taxa present in the U.S. This species was originally described by Murrill in 1902, was contested by many subsequent reports and synonymized with *G*. *lucidum* sensu lato or *G*. *resinaceum* [1, 6, 8–12]. Based on the presented phylogeny, *G*. *sessile* is distinct from *G*. *lucidum* sensu stricto, as well as *G*. *resinaceum*. *Ganoderma sessile* is one of the most widely distributed species of the laccate *Ganoderma* in the eastern U.S. Based on our collections in the U.S., we suspect that *G*. *sessiliforme* Murrill, a species originally described from Mexico [15], is either a synonym of *G*. *sessile*, or the geographic limits for this species occur south of the United States [27, 54]. The other two species in the “resinaceum subclade” are the Asian species *G*. *sichuanense* and *G*. cf. *weberianum*.

Based on the holotype morphology and ITS sequence (JQ781877), *G*. *sichuanense* was incorrectly labeled and is actually part of the *G*. *weberianum* species complex [2, 55]. The holotype of *G*. *sichuanense* (HMAS 42798) is from Sichuan, China, and is a sessile fruiting body with contextual chlamydospores, which are not characteristics of the widely cultivated Asian taxon, now recognized as *G*. *lingzhi* [49]. This morphology is highly similar to collections from south Florida that we labeled as *G*. cf. *weberianum*. The species *G*. *weberianum* was originally described from Pacific Islands with likely distribution in tropical locations in Africa and the Americas [56]. Later, Bazzalo & Wright [17] described *G*. *subamboinense* var. *laevisporum* as a morphologically similar species to *G*. *weberianum*, but from South America, including Argentina and Cuba. Unfortunately, due to a lack of sequences for other members of the *G*. *weberianum/subamboinense* species complex, we were unable to confidently determine an appropriate name for the collections made from south Florida. However, based on our phylogeny, the North American collections of *G*. cf. *weberianum* represent a well-supported lineage distinguished from the Asian collections, including the holotype labeled as *G*. *sichuanense*. Further investigations into this species complex are warranted, and it is likely that the collections of *G*. cf. *weberianum* from the US reported in this study are an undescribed species. The ITS sequences of *G*. *sichuanense*, *G*. *subamboinense and G*. *weberianum* from Genbank and the *G*. cf. *weberianum* from Florida are only 2–5 bases different. We suspect that sequencing from other loci, particularly *tef1α* and *rpb1*, may help to clearly distinguish these species.

Given the large geographic distributions of *G*. *oregonense* in the western U.S. and *G*. *tsugae* in the eastern U.S., we suspect that these sister taxa are native to the U.S. Both *G*. *oregonense* and *G*. *tsugae* have distinctly white context tissue, “rough” basidiospores, and are predominately associated with decay of conifers. In the eastern U.S., *G*. *tsugae* is geographically widespread in temperate eastern hemlock forests. Similarly, *G*. *oregonense* is geographically widespread in the western U.S. in the temperate forests dominated by conifers such as *Tsuga heterophylla*, *Pseudotsuga menziesii*, *Picea* spp., and *Abies* spp. The two species can be distinguished based on geographic location and size of basidiospores. The basidiospores of *G*. *oregonense* are slightly larger (12.9±1.7 x 8.0±1.3 μm) than those of *G*. *tsugae* (9.9±1.4 x 6.5±1.2 μm). Ecologically, *G*. *oregonense* and *G*. *tsugae* appear to be highly similar, but have been evolving independently due to geological barriers (e.g. the midwestern plains and Rocky Mountain range). However, we made four collections of *G*. *tsugae* from the southwestern United States (Arizona and New Mexico) from *Abies concolor* or *Pseudotsuga menziesii*. In Gilbertson & Ryvarden’s “North American Polypores” [1] they discuss collections of *G*. *tsugae* from Arizona and southern California exclusively on *Abies concolor* except for one collection from *Pseudotsuga menziesii*. Our collections from New Mexico on *Abies concolor* and *Pseudotsuga menziesii* are the first reports of *G*. *tsugae* from New Mexico. The populations of *G*. *tsugae* in the eastern U.S. and southwestern U.S. require further study to elucidate differences between the two disjunct populations.

Clade B included *G*. *lucidum* sensu stricto, *G*. *oregonense*, and *G*. *tsugae*. All three of these species are predominately found in temperate climates and all were found in our samples from the United States, although we strongly suspect *G*. *lucidum* sensu stricto is not native to the U.S. This clade was the least resolved with ITS alone, where none of these species were statistically separated. However, the phylogeny built with *tef1α* resolved the three species. Collections of *G*. *lucidum* sensu stricto were only found in two small distinct geographic ranges of northern Utah and northern California. Most subclades supported a general vicariant speciation hypothesis and we suspect that given the limited geographic distributions and the presented phylogenetic anomaly of the two populations, they are the result of two independent introductions of a European taxon to the U.S. This likely occurred by the production of the introduced fungus in field sites to produce basidiomata for medicinal purposes. In a similar case, the non-laccate European species, *G*. *adspersum* (Schulzer) Donk, has been introduced to California on popular European root stocks of almonds [57]. This species is causing tree failures, and economic losses to the California almond industry in the San Joaquin Valley [57]. Morphologically, *G*. *lucidum* sensu stricto in the U.S. can be diagnosed as having a true stipe, concentric growth zones in the context tissue, possessing a highly lacquered pileus and stipe, and being found on hardwood substrates. This morphological diagnosis is similar to collections of this taxon from Europe [2]. Further collections and genetic work will continue to elucidate the ecology and phylogenetic history of this species in the U.S. If this is indeed an introduced species, isolates of *G*. *lucidum* sensu lato of unknown origin should not be used outside for medicinal mushroom production since it is likely that these exotic species can become established. Furthermore, although it has not been widely documented, successful mating tests of European and U.S. collections of some *Ganoderma* species suggests that hybrids are possible. Theoretically, hybrids could produce more fit *Ganoderma* species that could potentially displace endemic *Ganoderma* species in North America, and the ramifications of this are not known.

Clade C, the most basal clade in the core *Ganoderma* included *G*. *boninense* from Asia and *G*. *zonatum* from the U.S. Similar to the other clades, these two species are sister to one another and geographically distant [2]. Both species are primary decay agents of woody monocots, especially palms. *Ganoderma boninense* is a primary decay fungus associated with palms, especially oil palm (*Elaeis guineensis*), in southeast Asia [2, 58]. Basal stem rot, caused by *G*. *boninense*, is the most significant disease of the economically important oil palm in southeast Asia [59]. Similar to *G*. *boninense*, *G*. *zonatum* is also considered a devastating pathogen and is associated with butt rot of palm trees in the Gulf South and along the Atlantic Coast in the southeastern U.S [60]. The geographic distribution of *G*. *zonatum* is likely similar to the geographic distribution of *Sabal palmetto* [61]. This is presumably due to a coevolutionary relationship. The elongated spores and dark brown context tissue of *G*. *boninense* and *G*. *zonatum* are the primary morphological diagnostic traits. Our study also revealed *Ganoderma zonatum* in North Carolina for the first time. This was found on a planted *Sabal palmetto* outside its natural range, which implies the possible movement of *G*. *zonatum* through the nursery trade. Our results suggest these species share a common ancestor and evolved into distinct species vicariously. The distribution of palms in other parts of the world may show other taxa in this clade. Through our collections and surveys, *G*. *zonatum* was the primary decay fungus associated with the decay of palms and bamboo, although there were other Ganodermataceae taxa occasionally found on palms. *Tomophagus colossus* was collected from a decaying palm in Gainesville, FL, and a non-laccate *Ganoderma* species similar to *G*. *tornatum* (*G*. *australe* species complex) was collected from decaying palm stumps throughout Florida.

Although the majority of collections in this survey were from the eastern U.S., we feel these data have captured a majority of the diversity of the laccate *Ganoderma* species that are found in the United States. This work also serves as a baseline to evaluate future *Ganoderma* collections. More collecting and analyses in the western United States could yield more diversity. For example, Murrill described *G*. *nevadense* from Nevada and *G*. *sequoiae* from California, both collected on conifers [7]. In our survey, four collections from conifer substrates from New Mexico and Arizona were found to be conspecific to collections of *G*. *tsugae* from the eastern U.S., but it is possible that with more sequencing some of these southwestern populations of *G*. *tsugae* could represent unique species similar to what was described by Murrill on conifers in the southwestern U.S [7].

Diagnosing species accurately is critical for studying the biology and ecology of fungi. Diagnostic characters such as DNA sequence, context tissue features, chlamydospores and geography were important for accurate species identification within the laccate *Ganoderma* species present in the U.S. From the 507 samples studied, thirteen taxa and twelve species of the Ganodermataceae are present in the United States and include: *G*. *curtisii*, *G*. *curtisii* f.sp. *meredithiae*, *G*. *lucidum* sensu stricto, *G*. *martinicense*, *G*. *oregonense*, *G*. *polychromum*, *G*. *ravenelii*, *G*. *sessile*, *G*. *tsugae*, *G*. *tuberculosum*, *G*. cf. *weberianum*, *G*. *zonatum*, and *Tomophagus colossus*. This study has unraveled some of the taxonomic difficulties associated with the laccate *Ganoderma* species that were once all considered as *G*. *lucidum* sensu lato. For example, all FLAS and NCSCLG herbarium collections collected from the U.S. and labeled as *G*. *lucidum* were not *G*. *lucidum* sensu stricto, and have been reannotated to one of the following species: *G*. *curtisii*, *G*. *martinicense*, *G*. *sessile* and *G*. *tuberculosum*. In addition, the presented data showed the ITS region, which is the most widely-used and important fungal barcode, is diagnostic for most species and subclades, but some species, especially in Clade B are not resolved. The *tef1α* locus captured more genetic diversity and is better at diagnosing the laccate *Ganoderma* to species level. However, there are more ITS sequences in GenBank compared to *tef1α*, and we recommend using both ITS and *tef1α* to avoid missing species with fewer accessioned sequences. As genome sequencing becomes more affordable, phylogenomics will be the most robust method to understand evolutionary patterns and mechanisms of speciation, which will undoubtedly resolve the Ganodermataceae. Further studies into the evolutionary history of the laccate *Ganoderma* species in the United States will elucidate important ecological relationships and more detailed geographic distribution patterns. Furthermore, sequencing more loci should be the focus of future studies, and could potentially resolve more cryptic species present in the U.S.

## Supporting information

S1 Table Isolates of the laccate *Ganoderma* from the United States with GenBank Accession numbers for ITS, *tef1α*, *rpb1*, and *rpb2* and other relevant collection data if present. (DOCX)
