# Supplementary material for: Elucidating "lucidum": Distinguishing the diverse laccate Ganoderma species of the United States
Source: PLoS One. 2018 Jul 18;13(7):e0199738. doi: 10.1371/journal.pone.0199738 (PMC6051579; doi:10.1371/journal.pone.0199738)
Supplement: S1 Table — (DOCX) [file pone.0199738.s001.docx]

**Supplementary Table 1**. Isolates of the laccate *Ganoderma* from the United States with GenBank Accession numbers for ITS, *tef1α, rpb1,* and *rpb2* and other relevant collection data if present.

|  | **GenBank Accession Numbers** | | | |  |  |  |  |  |  |
| --- | --- | --- | --- | --- | --- | --- | --- | --- | --- | --- |
| **Sample#** | **ITS** | ***tef1*α** | ***rpb1*** | ***rpb2*** | **Taxon** | **Year** | **City** | **State** | **Tree species** | **Tree Group** |
| 101SC | MG654303 |  |  |  | *G. sessile* | 2015 | Hiltonhead | SC | Quercus phellos | hardwood |
| 102NC | MG654074 | MG754727 |  | MG754851 | *G. curtisii* | 2015 | Charlotte | NC | Prunus cerasifera | hardwood |
| 103SC | MG654304 |  |  |  | *G. sessile* | 2015 | Clover | SC | Quercus nigra | hardwood |
| 105FL | MG654146 |  |  |  | *G. curtisii* | 2016 | Gainesville | FL | Quercus laurifolia | hardwood |
| 106GA | MG654305 |  |  |  | *G. sessile* | 2016 | Valdosta | GA | Carya illinoensis | hardwood |
| 110FL | MG654147 |  |  |  | *G. curtisii* | 2016 | Gainesville | FL | unknown stump (celtis?) | hardwood |
| 111TX | MG654306 | MG754747 | MG754816 | MG754866 | *G. sessile* | 2015 | Longview | TX | shipped with pine needles |  |
| 113FL | MG654307 | MG754748 |  | MG754867 | *G. sessile* | 2016 | Altamonte Springs | FL | Quercus nigra | hardwood |
| 114FL | MG654308 |  |  |  | *G. sessile* | 2016 | Micanopy | FL | Carya illinoensis | hardwood |
| 117TX | MG654309 | MG754749 | MG754817 | MG754868 | *G. sessile* | 2016 | Nagadoches | TX | Winged elm | hardwood |
| 118SC | MG654310 |  |  |  | *G. sessile* | 2016 | Clemson | SC | Quercus alba | hardwood |
| 121LA | MG654311 |  |  |  | *G. sessile* |  | Baton Rouge | LA | water oak removed 5 yrs. Prior | hardwood |
| 123FL | MG654416 | MG754774 | MG754841 |  | *G. zonatum* | 2016 | Pompano Beach | FL | dead palm | monocot |
| 124FL | MG654188 | MG754734 | MG754805 | MG754861 | *G. curtisii f.sp. meredithiae* | 2016 | Gainesville | FL | oak root near pine | conifer |
| 136FL | MG654148 |  | MG754783 | MG754852 | *G. curtisii* | 2016 | Sarasota | FL | Bambusa vulgaris | monocot |
| 140FL | MG654187 |  |  |  | *G. curtisii f.sp. meredithiae* | 2016 | Orlando | FL | Slash pine | conifer |
| 143FL | MG654206 |  |  |  | *G. ravenelii* | 2016 | Tallahassee | FL | *Quercus* | hardwood |
| 149TX | MG654149 |  |  |  | *G. curtisii* | 2016 | Nagadoches | TX |  |  |
| 150FL | MG654207 |  |  |  | *G. ravenelii* | 2016 | Hawthorne | FL |  |  |
| 151FL | MG654208 |  | MG754812 |  | *G. ravenelii* | 2016 | Hawthorne | FL |  |  |
| 158FL | MG654150 |  |  |  | *G. curtisii* | 2016 | Gainesville | FL | Poplar stump | hardwood |
| 160NC | MG654151 |  |  |  | *G. curtisii* | 2016 | Charlotte | NC | White oak | hardwood |
| 162FL | MG654152 |  |  |  | *G. curtisii* | 2016 | Milton | FL | white oak | hardwood |
| 164GA | MG654153 |  |  |  | *G. curtisii* | 2016 | Athens | GA | Southern red oak | hardwood |
| 165MO | MG654312 |  | MG754818 |  | *G. sessile* | 2016 | St. Louis Co. | MO | Quercus sp. | hardwood |
| 166MO | MG654313 |  |  |  | *G. sessile* | 2016 | St. Louis Co. | MO | Quercus sp. | hardwood |
| 167MO | MG654314 |  |  |  | *G. sessile* | 2016 | St. Louis Co. | MO | Quercus sp. | hardwood |
| 168MO | MG654315 |  |  |  | *G. sessile* | 2016 | St. Louis Co. | MO | Quercus sp. | hardwood |
| 171FL | MG654316 |  | MG754819 |  | *G. sessile* | 2016 | Miami-Dade County | FL | Quercus virginiana | hardwood |
| 175FL | MG654366 |  |  |  | *G. tuberculosum* | 2016 | Key Largo | FL | black olive | hardwood |
| 177NC | MG654154 |  | MG754784 | MG754853 | *G. curtisii* | 2016 | Wade | NC | Burned pine forest (terrestrially) | hardwood |
| 178NC | MG654155 |  |  |  | *G. curtisii* | 2016 | Wade | NC | hardwood stump | hardwood |
| 179NC | MG654417 | MG754775 | MG754842 | MG754875 | *G. zonatum* | 2016 | Wade | NC | Sabal palmetto | monocot |
| 193FL | MG654156 |  |  |  | *G. curtisii* | 2016 | Gainesville | FL |  |  |
| 194FL | MG654075 |  |  |  | *G. curtisii* | 2016 | Gainesville | FL |  |  |
| 195NC | MG654157 |  |  |  | *G. curtisii* | 2016 | Charlotte | NC | oak and pine stumps | hardwood |
| 196NC | MG654158 |  |  |  | *G. curtisii* | 2016 | Charlotte | NC | red oak stump | hardwood |
| 197NC | MG654159 |  |  |  | *G. curtisii* | 2016 | Charlotte | NC | oak stump | hardwood |
| 198NC | MG654160 |  |  |  | *G. curtisii* | 2016 | Charlotte | NC | terestriallly (buried roots) |  |
| 200MO | MG654317 |  |  |  | *G. sessile* | 2016 | Brussels | IL | oak stump | hardwood |
| 206NC | MG654161 |  |  |  | *G. curtisii* | 2016 | Charlotte | NC | Acer rubrum | hardwood |
| 207NC | MG654162 |  |  |  | *G. curtisii* | 2016 | Charlotte | NC | Quercus falcata | hardwood |
| 209FL | MG654163 |  |  |  | *G. curtisii* | 2016 | Gainesville | FL | Quercus shumardii | hardwood |
| 210FL | MG654318 |  |  |  | *G. sessile* | 2016 | Thonotosassa | FL | hardwood stump | hardwood |
| 211FL | MG654418 |  |  |  | *G. zonatum* | 2016 | Thonotosassa | FL | palm stump | monocot |
| 212FL | MG654164 |  |  |  | *G. curtisii* | 2016 | Tarpon Springs | FL | near sand live oak | hardwood |
| 213FL | MG654165 |  |  |  | *G. curtisii* | 2016 | Tarpon Springs | FL | hardwood stump | hardwood |
| 215TX | MG654166 |  |  |  | *G. curtisii* | 2016 | Kountze | TX | buried hardwood roots | hardwood |
| 218FL | MG654374 |  |  |  | *G. zonatum* | 2016 | Sarasota | FL | Bambusa vulgaris | monocot |
| 219FL | MG654419 |  |  |  | *G. zonatum* | 2016 | Tampa | FL | dead cabbage palm | monocot |
| 220FL | MG654420 |  |  |  | *G. zonatum* | 2016 | Tampa | FL | dead cabbage palm | monocot |
| 221FL | MG654421 |  |  |  | *G. zonatum* | 2016 | Tampa | FL | dead cabbage palm | monocot |
| 222FL | MG654422 |  |  |  | *G. zonatum* | 2016 | Tampa | FL | dead cabbage palm | monocot |
| 223FL | MG654167 |  | MG754785 | MG754854 | *G. curtisii* | 2016 | Tampa | FL | near declining laurel oak | hardwood |
| 224FL | MG654168 |  |  |  | *G. curtisii* | 2016 | Thonotosassa | FL | laurel oak | hardwood |
| 228DC | MG654319 | MG754750 | MG754820 | MG754869 | *G. sessile* | 2016 | Washington D.C. | VA | American elm | hardwood |
| 229FL | MG654423 |  |  |  | *G. zonatum* | 2016 | Boca Raton | FL | near dead black olive |  |
| 230GA | MG654174 |  |  |  | *G. martinicense* | 2016 | Athens | GA | oak | hardwood |
| 231NC | MG654182 | MG754736 | MG754801 |  | *G. martinicense* | 2016 | Denver | NC | honeylocust | hardwood |
| 232GA | MG654183 |  |  |  | *G. martinicense* | 2016 | Athens | GA | oak | hardwood |
| 233FL | MG654367 |  |  | MG754873 | *G. tuberculosum* | 2016 | Davie | FL | Albizia sp. | hardwood |
| 234FL | MG654169 |  |  |  | *G. curtisii* | 2016 | Gainesville | FL | Quercus sp. | hardwood |
| 235TX | MG654184 |  |  |  | *G. martinicense* | 2016 | Nagadoches | TX | terestrially |  |
| 236TX | MG654076 |  |  |  | *G. curtisii* | 2016 | Nagadoches | TX | oak snag | hardwood |
| 237TX | MG654170 |  |  |  | *G. curtisii* | 2016 | Nagadoches | TX | oak snag | hardwood |
| 238FL | MG654171 |  | MG754786 | MG754855 | *G. curtisii* | 2016 | Gainesville | FL | live oak | hardwood |
| 241FL | MG654077 |  |  |  | *G. curtisii* | 2016 | Gainesville | FL | dead stump |  |
| 246TX | MG654185 | MG754737 | MG754802 | MG754858 | *G. martinicense* | 2016 | Nagadoches | TX | terestrially |  |
| 248NC | MG654186 |  |  |  | *G. martinicense* | 2016 | Charlotte | NC | crabapple | hardwood |
| 249NC | MG654172 |  |  |  | *G. curtisii* | 2016 | Charlotte | NC | live oak | hardwood |
| 251FL | MG654424 |  |  |  | *G. zonatum* | 2016 | Gainesville | FL | Sabal palmetto | monocot |
| 252FL | MG654173 |  |  |  | *G. curtisii* |  |  |  |  |  |
| 253FL | MG654425 |  |  |  | *G. zonatum* | 2016 | Gainesville | FL |  | monocot |
| 254FL | MG654426 |  |  |  | *G. zonatum* | 2016 |  |  | Sabal palmetto | monocot |
| 255FL | MG654427 |  |  |  | *T. colossus* | 2016 | Miami | FL | Macrozamia mooreyi | cycad |
| 257FL | MG654078 |  |  |  | *G. curtisii* | 2016 | Micanopy | FL | near dead oak | hardwood |
| 261FL | MG654370 |  |  |  | *G. curtisii* | 2016 | Gainesville | FL | declining oak | hardwood |
| 262FL | MG654351 |  |  |  | *G. tuberculosum* | 2016 | Miami Lakes | FL | Ficus sp. | hardwood |
| 263FL | MG654079 |  |  |  | *G. curtisii* | 2016 | Miami Lakes | FL | live oak removed | hardwood |
| 265FL | MG654375 |  |  |  | *G. zonatum* | 2016 | Gainesville | FL | Sabal palmetto | monocot |
| 266FL | MG654080 |  |  |  | *G. curtisii* | 2016 | High Springs | FL | Declining oak | hardwood |
| 267FL | MG654081 |  |  |  | *G. curtisii* | 2016 | Gainesville | FL | Darlington Oak | hardwood |
| 268TX | MG654212 |  |  |  | *G. sessile* | 2016 | Alpine | TX | Quercus buckleyi | hardwood |
| 269TX | MG654352 |  |  |  | *G. tuberculosum* | 2016 | Brownsville | TX | Mesquite | hardwood |
| 270NC | MG654175 |  |  |  | *G. martinicense* | 2016 | Charlotte | NC | Prunus sp. | hardwood |
| 271NC | MG654213 |  |  |  | *G. sessile* | 2016 | Charlotte | NC | Crabapple | hardwood |
| 281LA | MG654214 |  |  |  | *G. sessile* | 2016 | Thibodaux | LA | Water oak (living) | hardwood |
| 298NJ | MG654215 |  |  |  | *G. sessile* | 2017 | Ridgewood | NJ | Prunus sp. | hardwood |
| 300FL | MG654082 |  |  |  | *G. curtisii* | 2017 | Gainesville | FL | Quercus hemisphaerica | hardwood |
| 302NC | MG654083 |  |  |  | *G. curtisii* | 2017 | Raleigh | NC | Prunus sp. | hardwood |
| 305FL | MG654376 |  |  |  | *G. zonatum* | 2017 | Dunnellon | FL | Sabal palmetto | monocot |
| 306FL | MG654216 |  |  |  | *G. sessile* | 2017 | Micanopy | FL | Fagaceae | hardwood |
| 308FL | MG654217 |  |  |  | *G. sessile* |  | Gainesville | FL | Quercus hemisphaerica | hardwood |
| 309FL | MG654084 |  |  |  | *G. curtisii* |  | Gainesville | FL | Celtis laevigata |  |
| 310FL | MG654085 |  |  |  | *G. curtisii* |  | Citra | FL | Persea borbonia |  |
| 311FL | MG654086 |  |  |  | *G. curtisii* |  |  | FL |  |  |
| 317UT | MG654218 | MG754751 |  |  | *G. sessile* |  | St. George | UT |  | hardwood |
| 324FL | MG654353 | MG754767 |  |  | *G. tuberculosum* |  |  |  |  |  |
| 325NJ | MG654219 |  |  |  | *G. sessile* |  | Ridgewood | NJ | Quercus sp. | hardwood |
| 326NY | MG654220 |  |  |  | *G. sessile* |  | Peekskill | NY | Fagus americana | hardwood |
| 327NY | MG654087 |  |  |  | *G. curtisii* |  | Peekskill | NY | unknown |  |
| 330OR | MG654196 | MG754742 |  |  | *G. polychromum* | 2017 | Vancouver | WA |  |  |
| 331IL | MG654221 |  |  |  | *G. sessile* |  | Boiling Brook | IL | honeylocust | hardwood |
| 335FL | MG654354 | MG754768 |  |  | *G. tuberculosum* | 2017 | Fort Lauderdale | FL | Pongamia pinnata | hardwood |
| 340OR | MG654189 |  |  |  | *G. oregonense* |  |  | OR |  | conifer |
| BWH54E04MN | MG654222 |  |  |  |  |  |  |  |  |  |
| bwh58bFL | MG654088 |  |  |  |  |  |  |  |  |  |
| bwh58gFL | MG654377 |  |  |  |  |  |  |  |  |  |
| FLASF59206 | MG654355 |  |  |  |  |  |  |  |  |  |
| FLASF59210 | MG654371 |  |  |  |  |  |  |  |  |  |
| MS182AZ |  |  |  | MG754864 | *G. tsugae* |  |  | AZ |  | conifer |
| MS183CA |  | MG754723 | MG754798 |  | *G. lucidum* |  |  | CA |  | hardwood |
| MS187FL | MG654211 | MG754745 | MG754813 | MG754865 | *G. ravenelii* |  |  | FL |  | hardwood |
| MS188x | MG654320 | MG754752 |  | MG754870 | *G. sessile* |  |  | ? |  |  |
| MS337CA | MG654066 |  |  |  | *G. lucidum* |  |  | CA |  | hardwood |
| MS343OR | MG654197 | MG754743 |  |  | *G. polychromum* |  |  |  |  |  |
| GAN11 |  | MG754776 |  | MG754876 | *G. zonatum* |  | Broward County | FL |  | monocot |
| PLM540 | MG654368 |  |  |  | *G. tuberculosum* | 2011 | Broward County | FL | Albizia sp. | hardwood |
| PLM684 | MG654369 | MG754769 |  |  | *G. tuberculosum* | 2013 | Miami-Dade County | FL | Bursera simaruba | hardwood |
| UMN1 | MG654089 |  |  |  | *G. curtisii* |  |  |  |  |  |
| UMN2 | MG654223 |  |  |  | *G. sessile* |  |  |  |  |  |
| UMNAK1 | MG654190 | MG754740 | MG754808 |  | *G. oregonense* |  | Juneau | AK |  |  |
| UMNAK2 | MG654191 |  |  |  | *G. oregonense* |  | Juneau | AK |  |  |
| UMNAL2 | MG654176 |  |  |  | *G. martinicense* |  | Florence | AL |  |  |
| UMNAZ9 | MG654321 | MG754763 | MG754835 |  | *G. tsugae* |  | Flagstaff | AZ | white fir | conifer |
| UMNCA1 | MG654198 |  |  |  | *G. polychromum* |  |  | CA |  |  |
| UMNCA10 | MG654201 |  |  |  | *G. polychromum* |  | Stevenson | CA |  |  |
| UMNCA11 | MG654192 |  |  |  | *G. oregonense* |  | Albion | CA |  |  |
| UMNCA12 | MG654202 |  |  |  | *G. polychromum* |  | Petaluma | CA |  |  |
| UMNCA13 | MG654193 |  |  |  | *G. oregonense* |  | Nevada County | CA |  |  |
| UMNCA14 | MG654069 |  |  |  | *G. oregonense* |  | Berkley | CA |  |  |
| UMNCA6 | MG654067 | MG754724 |  |  | *G. oregonense* |  | Grass Valley | CA |  |  |
| UMNCA7 | MG654199 |  |  |  | *G. polychromum* |  | Watsonville | CA |  |  |
| UMNCA8 | MG654068 |  |  |  | *G. oregonense* |  | Penryn | CA |  |  |
| UMNCA9 | MG654200 |  |  |  | *G. sessile* |  | Los Angeles | CA |  |  |
| UMNDC1 | MG654090 |  |  |  | *G. curtisii* |  |  | DC |  |  |
| UMNFL1 | MG654378 |  |  |  | *G. zonatum* |  |  | FL |  |  |
| UMNFL10 | MG654227 | MG754753 | MG754821 |  | *G. sessile* |  |  | FL |  |  |
| UMNFL100 | MG654373 | MG754762 | MG754834 |  | *G. c.f. weberianum* | 2015 | Bradenton | FL | hardwood | hardwood |
| UMNFL103 | MG654407 |  | MG754846 |  | *G. zonatum* |  | Fort Myers | FL | Neodypsis decaryi | monocot |
| UMNFL104 | MG654238 |  | MG754824 |  | *G. sessile* | 2015 | Sarasota | FL | Casaurina sp. | hardwood |
| UMNFL105 | MG654408 | MG754780 | MG754847 |  | *G. zonatum* | 2015 | Mound St. Pal | FL |  |  |
| UMNFL107 | MG654113 |  |  |  | *G. curtisii f.sp. meredithiae* | 2015 |  | FL | South florida slash pine | conifer |
| UMNFL108 | MG654409 |  |  |  | *G. zonatum* | 2015 |  | FL | palm | monocot |
| UMNFL109 | MG654410 |  |  |  | *G. zonatum* | 2015 |  | FL | Saw palmetto | monocot |
| UMNFL11 | MG654228 |  |  |  | *G. sessile* |  |  | FL |  |  |
| UMNFL110 | MG654429 |  | MG754850 |  | *T. colossus* |  | Lido Key | FL | Casaurina wood chips | hardwood |
| UMNFL112 | MG654411 |  |  |  | *G. zonatum* |  | Long boat key | FL | palm | monocot |
| UMNFL114 | MG654358 |  |  |  | *G. tuberculosum* |  | Coon Key | FL | Casaurina sp. | hardwood |
| UMNFL117 | MG654359 | MG754771 |  |  | *G. tuberculosum* |  | Lido Key | FL | Casaurina sp. | hardwood |
| UMNFL12 | MG654380 |  |  |  | *G. zonatum* |  |  | FL |  |  |
| UMNFL124 | MG654430 |  |  |  | *T. colossus* |  | Lido Key | FL | Casaurina sp. | hardwood |
| UMNFL125 | MG654239 | MG754755 | MG754825 |  | *G. sessile* |  | Myakka | FL | Quercus sp. | hardwood |
| UMNFL127 | MG654240 |  |  |  | *G. sessile* |  | Myakka | FL | unknown hardwood | hardwood |
| UMNFL128 | MG654241 |  | MG754826 |  | *G. sessile* |  | Myakka | FL | hardwood | hardwood |
| UMNFL129 | MG654242 |  |  |  | *G. sessile* |  | Myakka | FL | unknown hardwood | hardwood |
| UMNFL13 | MG654094 |  |  |  | *G. curtisii* |  |  | FL |  |  |
| UMNFL131 | MG654412 |  |  |  | *G. zonatum* |  | Myakka | FL | palm | monocot |
| UMNFL132 | MG654243 |  |  |  | *G. sessile* |  | Myakka | FL | Quercus sp. | hardwood |
| UMNFL135 | MG654244 |  |  |  | *G. sessile* |  | Myakka | FL | Quercus sp. | hardwood |
| UMNFL136 | MG654245 |  |  |  | *G. sessile* |  | Myakka | FL | Quercus sp. | hardwood |
| UMNFL137 | MG654413 | MG754781 | MG754848 |  | *G. zonatum* |  | Myakka | FL | palm | monocot |
| UMNFL14 | MG654229 |  |  |  | *G. sessile* |  |  | FL |  |  |
| UMNFL140 | MG654246 |  |  |  | *G. sessile* |  | Myakka | FL | Quercus sp. | hardwood |
| UMNFL141 | MG654247 |  |  |  | *G. sessile* |  | Myakka | FL | unknown hardwood | hardwood |
| UMNFL142 | MG654248 |  |  |  | *G. sessile* |  | Myakka | FL | Quercus sp. | hardwood |
| UMNFL143 | MG654249 |  |  |  | *G. sessile* |  | Myakka | FL | Quercus sp. | hardwood |
| UMNFL144 | MG654250 |  |  |  | *G. sessile* |  | Myakka | FL | Quercus sp. | hardwood |
| UMNFL145 | MG654251 |  |  |  | *G. sessile* |  | Myakka | FL | Quercus sp. | hardwood |
| UMNFL146 | MG654360 | MG754772 |  |  | *G. tuberculosum* |  | Sebring | FL |  | hardwood |
| UMNFL147 | MG654114 |  |  |  | *G. curtisii* |  | Jacksonville | FL |  |  |
| UMNFL15 | MG654095 |  |  |  | *G. curtisii* |  |  | FL |  |  |
| UMNFL150 | MG654209 |  |  |  | *G. curtisii* |  | Hudson | FL |  |  |
| UMNFL151 | MG654431 |  |  |  | *T. colossus* |  | Murdock | FL |  |  |
| UMNFL152 | MG654115 |  |  |  | *G. curtisii* |  | Hudson | FL |  |  |
| UMNFL153 | MG654210 |  |  |  | *G. ravenelii* |  | Hudson | FL |  |  |
| UMNFL156 | MG654361 |  |  |  | *G. tuberculosum* | 2016 |  | FL | Citrus sp. | hardwood |
| UMNFL157 | MG654362 |  |  |  | *G. tuberculosum* | 2016 |  | FL | Citrus sp. | hardwood |
| UMNFL158 | MG654363 |  |  |  | *G. tuberculosum* | 2016 |  | FL | Citrus sp. | hardwood |
| UMNFL16 | MG654381 | MG754777 | MG754843 |  | *G. zonatum* |  |  | FL |  |  |
| UMNFL160 | MG654364 |  | MG754840 |  | *G. tuberculosum* | 2016 |  | FL | Citrus sp. | hardwood |
| UMNFL162 | MG654365 | MG754773 |  |  | *G. tuberculosum* | 2016 |  | FL | Citrus sp. | hardwood |
| UMNFL164 | MG654252 |  |  |  | *G. sessile* |  |  | FL | Cupaniopsis anacardioides | hardwood |
| UMNFL168 | MG654253 |  |  |  | *G. sessile* |  |  | FL | Cupaniopsis anacardioides | hardwood |
| UMNFL17 | MG654428 |  |  |  | *T. colossus* |  | Gainesville | FL | palm | monocot |
| UMNFL174 | MG654254 |  |  |  | *G. sessile* |  |  | FL | Cupaniopsis anacardioides | hardwood |
| UMNFL18 | MG654382 |  |  |  | *G. zonatum* |  |  | FL |  |  |
| UMNFL180 | MG654255 |  |  |  | *G. sessile* |  |  | FL | Cupaniopsis anacardioides | hardwood |
| UMNFL186 | MG654116 |  |  |  | *G. curtisii* |  |  | FL | Bambusa vulgarus | monocot |
| UMNFL187 |  |  | MG754814 |  | *G. ravenelii* | 2016 |  | FL |  | hardwood |
| UMNFL188 |  | MG754746 | MG754815 |  | *G. ravenelii* | 2016 |  | FL |  | hardwood |
| UMNFL19 | MG654230 | MG754754 | MG754822 |  | *G. sessile* |  |  | FL |  |  |
| UMNFL2 | MG654224 |  |  |  | *G. sessile* |  |  | FL |  |  |
| UMNFL20 | MG654231 |  | MG754823 |  | *G. sessile* |  |  | FL |  |  |
| UMNFL21 | MG654383 |  |  |  | *G. zonatum* |  |  | FL |  |  |
| UMNFL22 | MG654232 |  |  |  | *G. sessile* |  |  | FL |  |  |
| UMNFL23 | MG654096 |  | MG754787 |  | *G. curtisii* |  |  | FL |  |  |
| UMNFL24 | MG654384 |  |  |  | *G. zonatum* |  |  | FL |  |  |
| UMNFL25 | MG654356 |  |  |  | *G. tuberculosum* |  |  | FL |  |  |
| UMNFL26 | MG654233 |  |  |  | *G. sessile* |  |  | FL |  |  |
| UMNFL28 | MG654097 | MG754728 | MG754788 | MG754856 | *G. curtisii* |  | Gainesville | FL |  |  |
| UMNFL29 | MG654098 |  |  |  | *G. curtisii* |  |  | FL |  |  |
| UMNFL3 | MG654091 |  |  |  | *G. curtisii* |  |  | FL |  |  |
| UMNFL30 | MG654099 |  |  |  | *G. curtisii* |  |  | FL |  |  |
| UMNFL31 | MG654100 |  |  |  | *G. curtisii* |  |  | FL |  |  |
| UMNFL32 | MG654372 | MG754761 | MG754833 |  | *G. c.f. weberianum* |  | Davie | FL |  |  |
| UMNFL33 | MG654357 |  |  |  | *G. tuberculosum* |  | Maitland | FL |  |  |
| UMNFL35 | MG654234 |  |  |  | *G. sessile* |  |  | FL |  |  |
| UMNFL36 | MG654385 |  |  |  | *G. zonatum* |  |  | FL |  |  |
| UMNFL37 | MG654235 |  |  |  | *G. sessile* |  |  | FL |  |  |
| UMNFL39 | MG654101 |  |  |  | *G. curtisii* |  |  | FL |  |  |
| UMNFL4 | MG654092 |  |  |  | *G. curtisii* |  |  | FL |  |  |
| UMNFL41 | MG654386 |  |  |  | *G. zonatum* |  | Manatee County | FL |  |  |
| UMNFL43 | MG654387 |  |  |  | *G. zonatum* |  |  | FL |  |  |
| UMNFL44 | MG654388 |  |  |  | *G. zonatum* |  | Manatee County | FL |  |  |
| UMNFL46 | MG654236 |  |  |  | *G. sessile* | 2014 |  | FL |  |  |
| UMNFL47 | MG654389 |  |  |  | *G. zonatum* |  | Sarasota county | FL |  |  |
| UMNFL48 | MG654102 |  |  |  | *G. curtisii* |  | Bradenton | FL |  |  |
| UMNFL5 | MG654379 |  |  |  | *G. zonatum* |  |  | FL |  |  |
| UMNFL50 | MG654103 | MG754735 | MG754806 | MG754862 | *G. curtisii f.sp. meredithiae* |  | Sarasota county | FL | South florida slash pine | conifer |
| UMNFL51 | MG654390 |  |  |  | *G. zonatum* |  | Sarasota county | FL |  |  |
| UMNFL52 | MG654391 |  |  |  | *G. zonatum* |  |  | FL | Queen palm | monocot |
| UMNFL53 | MG654392 |  |  |  | *G. zonatum* |  | Fort Myers | FL | Saw palmetto | monocot |
| UMNFL54 | MG654393 |  |  |  | *G. zonatum* |  |  | FL | Sabal palmetto | monocot |
| UMNFL55 | MG654237 |  |  |  | *G. sessile* |  | Fakahatchee | FL |  |  |
| UMNFL56 | MG654104 |  |  |  | *G. curtisii* |  |  | FL | Quercus sp. | hardwood |
| UMNFL57 | MG654394 |  |  |  | *G. zonatum* |  | Lido Beach | FL | unknown palm | monocot |
| UMNFL59 | MG654395 |  |  |  | *G. zonatum* |  |  | FL | unknown palm | monocot |
| UMNFL6 | MG654093 |  |  |  | *G. curtisii* |  |  | FL |  |  |
| UMNFL60 | MG654105 | MG754729 | MG754789 |  | *G. curtisii* |  |  | FL |  |  |
| UMNFL63 | MG654396 |  |  |  | *G. zonatum* |  | Sarasota | FL |  |  |
| UMNFL64 | MG654106 |  | MG754807 | MG754863 | *G. curtisii f.sp. meredithiae* |  | Manatee County | FL |  |  |
| UMNFL65 | MG654397 |  |  |  | *G. zonatum* |  | Fort Myers | FL |  |  |
| UMNFL7 | MG654225 |  |  |  | *G. sessile* |  |  | FL |  |  |
| UMNFL70 | MG654398 |  |  |  | *G. zonatum* |  | Bradenton | FL | thatch palm | monocot |
| UMNFL74 | MG654399 |  |  |  | *G. zonatum* |  | Manatee County | FL | thatch palm | monocot |
| UMNFL75 | MG654400 |  |  |  | *G. zonatum* |  | Bradenton | FL | decaying palm | monocot |
| UMNFL77 | MG654107 |  |  |  | *G. curtisii* |  | Bradenton | FL | Quercus sp. | hardwood |
| UMNFL78 | MG654401 |  |  |  | *G. zonatum* |  | Sarasota | FL | Queen palm | monocot |
| UMNFL79 | MG654108 |  |  |  | *G. curtisii* |  |  | FL |  |  |
| UMNFL8 | MG654226 |  |  |  | *G. sessile* |  |  | FL |  |  |
| UMNFL80 | MG654109 | MG754730 | MG754790 |  | *G. curtisii* | 2015 | Alachua County | FL | Quercus shumardii | hardwood |
| UMNFL82 |  | MG754770 |  | MG754874 | *G. tuberculosum* |  |  | FL |  |  |
| UMNFL84 | MG654110 |  |  |  | *G. curtisii f.sp. meredithiae* | 2015 | Sarasota | FL | Slash pine | conifer |
| UMNFL85 | MG654402 | MG754778 | MG754844 | MG754877 | *G. zonatum* | 2015 |  | FL | Saw palmetto | monocot |
| UMNFL86 | MG654111 |  |  |  | *G. curtisii* | 2015 |  | FL |  |  |
| UMNFL87 | MG654112 |  |  |  | *G. curtisii* | 2015 |  | FL | Quercus sp. | hardwood |
| UMNFL89 | MG654403 | MG754779 | MG754845 |  | *G. zonatum* | 2015 |  | FL | palm | monocot |
| UMNFL91 | MG654404 |  |  |  | *G. zonatum* |  |  | FL | Phoenix canariensis | monocot |
| UMNFL93 | MG654405 |  |  |  | *G. zonatum* |  |  | FL | Queen palm | monocot |
| UMNFL99 | MG654406 |  |  |  | *G. zonatum* | 2015 | Bradenton | FL | palm | monocot |
| UMNGA1 | MG654117 | MG754731 | MG754791 | MG754857 | *G. curtisii* |  |  | GA |  |  |
| UMNGA2 | MG654118 |  | MG754792 |  | *G. curtisii* |  |  | GA |  |  |
| UMNGA3 | MG654119 |  |  |  | *G. curtisii* |  |  | GA |  |  |
| UMNGA4 | MG654120 |  |  |  | *G. curtisii* |  |  | GA |  |  |
| UMNGA5 | MG654121 |  |  |  | *G. curtisii* |  |  | GA |  |  |
| UMNGA6 | MG654122 |  |  |  | *G. curtisii* |  |  | GA |  |  |
| UMNGA7 | MG654123 |  |  |  | *G. curtisii* |  |  | GA |  |  |
| UMNIL1 | MG654124 |  |  |  | *G. curtisii* |  |  | IL |  |  |
| UMNIN1 | MG654256 |  |  |  | *G. sessile* |  |  | IN |  |  |
| UMNKY1 | MG654257 | MG754756 | MG754827 |  | *G. sessile* |  |  | KY |  |  |
| UMNKY2 | MG654323 |  |  |  | *G. tsugae* |  |  | KY | Hemlock | conifer |
| UMNLA1 | MG654125 |  | MG754793 |  | *G. curtisii* |  |  | LA |  |  |
| UMNMA1 | MG654258 |  |  |  | *G. sessile* |  |  | MA |  |  |
| UMNMA2 | MG654259 |  |  |  | *G. sessile* |  |  | MA |  |  |
| UMNMA3 | MG654126 |  |  |  | *G. curtisii* |  |  | MA |  |  |
| UMNMI1 | MG654260 |  |  |  | *G. sessile* |  |  | MI |  |  |
| UMNMI13 | MG654262 |  |  |  | *G. sessile* |  | Grand Junction | MI |  |  |
| UMNMI14 | MG654263 |  |  |  | *G. sessile* |  | Royal Oak | MI | maple | hardwood |
| UMNMI15 | MG654264 |  |  |  | *G. sessile* |  | Detroit | MI |  |  |
| UMNMI16 | MG654265 |  |  |  | *G. sessile* |  | Harpers wood | MI | silver maple | hardwood |
| UMNMI17 | MG654266 |  |  |  | *G. sessile* |  | Detroit | MI | crabapple | hardwood |
| UMNMI18 | MG654267 |  |  |  | *G. sessile* |  | Fomdale | MI | silver maple | hardwood |
| UMNMI19 | MG654268 |  |  |  | *G. sessile* |  | Fomdale | MI | oak | hardwood |
| UMNMI2 | MG654261 |  |  |  | *G. sessile* |  |  | MI |  |  |
| UMNMI20 | MG654324 | MG754764 | MG754836 |  | *G. tsugae* |  | Dyer Island | MI | Hemlock | conifer |
| UMNMI21 | MG654325 |  |  |  | *G. tsugae* |  |  | MI | birch | hardwood |
| UMNMI22 | MG654269 | MG754757 | MG754828 |  | *G. sessile* |  | Fomdale | MI |  |  |
| UMNMI23 | MG654270 |  |  |  | *G. sessile* |  | Royal Oak | MI |  |  |
| UMNMI24 | MG654271 | MG754758 | MG754829 |  | *G. sessile* |  | Royal Oak | MI |  |  |
| UMNMI25 | MG654272 |  |  |  | *G. sessile* |  | Shores St. Claire | MI | maple | hardwood |
| UMNMI26 | MG654127 |  |  |  | *G. curtisii* |  | Grand Junction | MI | maple | hardwood |
| UMNMI27 | MG654273 |  |  |  | *G. sessile* |  | Royal Oak | MI | oak | hardwood |
| UMNMI28 | MG654274 |  |  |  | *G. sessile* |  | Royal Oak | MI | maple | hardwood |
| UMNMI29 | MG654275 |  |  |  | *G. sessile* |  | Southfield | MI |  |  |
| UMNMI30 | MG654326 |  | MG754837 | MG754871 | *G. tsugae* |  | Colonial | MI | Hemlock | conifer |
| UMNMN2 | MG654276 |  |  |  | *G. sessile* |  |  | MN |  |  |
| UMNMN28 | MG654283 |  |  |  | *G. sessile* |  | St. Paul | MN |  |  |
| UMNMN3 | MG654277 |  | MG754794 |  | *G. sessile* |  |  | MN |  |  |
| UMNMN4 | MG654278 |  | MG754838 |  | *G. sessile* |  |  | MN |  |  |
| UMNMN5 | MG654279 |  |  |  | *G. sessile* |  |  | MN |  |  |
| UMNMN6 | MG654280 |  |  |  | *G. sessile* |  |  | MN |  |  |
| UMNMN7 | MG654327 |  |  |  | *G. tsugae* |  |  | MN | Hemlock | conifer |
| UMNMN8 | MG654281 |  |  |  | *G. sessile* |  |  | MN |  |  |
| UMNMN9 | MG654282 |  |  |  | *G. sessile* |  | Aspen | MN |  |  |
| UMNMO1 | MG654284 |  |  |  | *G. sessile* |  |  | MO |  |  |
| UMNMO2 | MG654285 |  |  |  | *G. sessile* |  |  | MO |  |  |
| UMNMO3 | MG654128 |  |  |  | *G. curtisii* |  |  | MO |  |  |
| UMNNC1 | MG654129 |  |  |  | *G. curtisii* |  |  | NC |  |  |
| UMNNC2 | MG654328 |  |  |  | *G. tsugae* |  |  | NC | Hemlock | conifer |
| UMNNC3 | MG654130 | MG754732 |  |  | *G. curtisii* |  |  | NC |  |  |
| UMNNC4 | MG654329 | MG754765 |  | MG754872 | *G. tsugae* |  |  | NC | Hemlock | conifer |
| UMNNH1 | MG654131 |  | MG754795 |  | *G. curtisii* | 2016 |  | NH |  |  |
| UMNNH2 | MG654132 |  |  |  | *G. curtisii* | 2016 |  | NH |  |  |
| UMNNH3 | MG654330 |  |  |  | *G. tsugae* |  | Tamworth | NH | Hemlock | hardwood |
| UMNNJ1 | MG654133 |  |  |  | *G. curtisii* | 2015 |  | NJ |  |  |
| UMNNJ2 | MG654134 |  |  |  | *G. curtisii* |  |  | NJ |  |  |
| UMNNJ3 | MG654135 |  |  |  | *G. curtisii* |  |  | NJ |  |  |
| UMNNJ5 | MG654136 |  |  |  | *G. curtisii* |  |  | NJ |  |  |
| UMNNM1 | MG654286 |  |  |  |  |  |  |  |  |  |
| UMNNM13 | MG654331 |  | MG754839 |  | *G. tsugae* |  | Cloudcroft | NM | Douglas Fir | conifer |
| UMNNM46 | MG654332 |  |  |  | *G. tsugae* |  | Mescalero | NM | White fir | conifer |
| UMNNY1 | MG654287 |  |  |  | *G. sessile* |  |  | NY |  |  |
| UMNNY11 | MG654291 |  |  |  | *G. sessile* |  |  | NY |  |  |
| UMNNY12 | MG654292 |  |  |  | *G. sessile* |  |  | NY |  |  |
| UMNNY13 | MG654293 |  |  |  | *G. sessile* |  |  | NY |  |  |
| UMNNY14 | MG654294 |  | MG754830 |  | *G. sessile* |  |  | NY |  |  |
| UMNNY2 | MG654288 |  |  |  | *G. sessile* |  |  | NY |  |  |
| UMNNY3 | MG654289 |  |  |  | *G. sessile* |  | Watkins Glen | NY |  |  |
| UMNNY4 | MG654137 |  |  |  | *G. curtisii* |  |  | NY |  |  |
| UMNNY5 | MG654290 |  |  |  | *G. sessile* |  | Brooklyn | NY |  |  |
| UMNNY6 | MG654138 |  |  |  | *G. curtisii* |  | Babylon | NY |  |  |
| UMNOH1 | MG654295 |  |  |  | *G. sessile* |  |  | OH |  |  |
| UMNOH2 | MG654296 |  |  |  | *G. sessile* |  |  | OH |  |  |
| UMNOH3 | MG654297 |  |  |  | *G. sessile* |  |  | OH |  |  |
| UMNOH4 | MG654298 | MG754759 | MG754831 |  | *G. sessile* |  |  | OH |  |  |
| UMNOK1 | MG654139 |  |  |  | *G. curtisii* |  |  | OK |  |  |
| UMNOR1 | MG654194 | MG754741 | MG754809 |  | *G. oregonense* |  |  | OR |  |  |
| UMNOR2 | MG654203 |  |  |  | *G. polychromum* |  |  | OR |  |  |
| UMNOR3 | MG654204 | MG754744 | MG754810 |  | *G. polychromum* |  | Canyonville | OR |  |  |
| UMNPA1 | MG654140 |  | MG754796 |  | *G. curtisii* |  |  | PA |  |  |
| UMNSC1 | MG654299 |  |  |  | *G. sessile* |  |  | SC |  |  |
| UMNSC2 | MG654141 | MG754733 | MG754797 |  | *G. curtisii* |  |  | SC |  |  |
| UMNSC3 | MG654414 |  |  |  | *G. zonatum* |  |  | SC |  |  |
| UMNSC4 | MG654415 | MG754782 | MG754849 |  | *G. zonatum* |  |  | SC |  |  |
| UMNSC5 | MG654300 |  |  |  | *G. sessile* |  |  | SC |  |  |
| UMNSC6 | MG654301 |  |  |  | *G. sessile* |  |  | SC |  |  |
| UMNSC7 | MG654177 |  |  | MG754859 | *G. martinicense* |  |  | SC | Cactus | cactus |
| UMNSC8 | MG654142 |  |  |  | *G. curtisii* |  |  | SC |  |  |
| UMNSC9 | MG654143 |  |  |  | *G. curtisii* |  |  | SC |  |  |
| UMNTN1 | MG654178 | MG754738 | MG754803 | MG754860 | *G. martinicense* |  |  | TN |  |  |
| UMNTN2 | MG654179 |  |  |  | *G. martinicense* |  |  | TN |  |  |
| UMNTN3 | MG654180 | MG754739 |  |  | *G. martinicense* |  |  | TN |  |  |
| UMNTN4 | MG654181 |  |  |  | *G. martinicense* |  |  | TN |  |  |
| UMNTX1 | MG654144 |  |  |  | *G. curtisii f.sp. meredithiae* |  | Smithville | TX | Pinus sp. | conifer |
| UMNTX3 |  |  | MG754804 |  | *G. martinicense* |  | Nagadoches | TX |  |  |
| UMNUT1 | MG654070 | MG754725 | MG754799 |  | *G. "lucidum"* |  |  | UT | Oak | hardwood |
| UMNUT7 | MG654071 | MG754726 | MG754800 |  | *G. "lucidum"* |  | Farmington | UT |  |  |
| UMNUT8 | MG654072 |  |  |  | *G. "lucidum"* |  | Rivertun | UT |  |  |
| UMNUT9 | MG654073 |  |  |  | *G. "lucidum"* |  | Rivertun | UT |  |  |
| UMNVA1 | MG654145 |  |  |  | *G. curtisii* |  |  | VA | oak | hardwood |
| UMNWA1 | MG654195 |  | MG754811 |  | ***G. oregonense*** |  |  | **WA** |  |  |
| UMNWA2 | MG654205 |  |  |  | *G. polychromum* |  |  | WA |  |  |
| UMNWI1 | MG654333 |  |  |  | *G. tsugae* |  |  | WI | Hemlock | conifer |
| UMNWI10 | MG654337 |  |  |  | *G. tsugae* |  |  | WI | Hemlock | conifer |
| UMNWI12 | MG654338 |  |  |  | *G. tsugae* |  |  | WI | Hemlock | conifer |
| UMNWI13 | MG654339 |  |  |  | *G. tsugae* |  |  | WI | Hemlock | conifer |
| UMNWI14 | MG654340 |  |  |  | *G. tsugae* |  |  | WI | Hemlock | conifer |
| UMNWI16 | MG654341 |  |  |  | *G. tsugae* |  |  | WI | Hemlock | conifer |
| UMNWI17 | MG654342 |  |  |  | *G. tsugae* |  |  | WI | Hemlock | conifer |
| UMNWI18 | MG654343 |  |  |  | *G. tsugae* |  |  | WI | Hemlock | conifer |
| UMNWI2 | MG654334 |  |  |  | *G. tsugae* |  |  | WI | Hemlock | conifer |
| UMNWI22 | MG654344 |  |  |  | *G. tsugae* |  |  | WI | Hemlock | conifer |
| UMNWI23 | MG654345 | MG754766 |  |  | *G. tsugae* |  |  | WI | Hemlock | conifer |
| UMNWI24 | MG654346 |  |  |  | *G. tsugae* |  |  | WI | Hemlock | conifer |
| UMNWI25 | MG654347 |  |  |  | *G. tsugae* |  |  | WI | Hemlock | conifer |
| UMNWI3 | MG654335 |  |  |  | *G. tsugae* |  |  | WI | Hemlock | conifer |
| UMNWI30 | MG654348 |  |  |  | *G. tsugae* |  |  | WI | Hemlock | conifer |
| UMNWI31 | MG654349 |  |  |  | *G. tsugae* |  |  | WI | Hemlock | conifer |
| UMNWI36 | MG654350 |  |  |  | *G. tsugae* |  | Madelin Island | WI | Hemlock | conifer |
| UMNWI4 | MG654336 |  |  |  | *G. tsugae* |  |  | WI | Hemlock | conifer |
| UMNWV1 | MG654302 | MG754760 | MG754832 |  | *G. sessile* |  |  | WV |  |  |
